# Phytochemical analysis, identification of bioactive compounds using GC-MS, *in vitro* and *in silico* hypoglycemic potential, *in vitro* antioxidant potential, and *in silico* ADME analysis of *Chlorophytum comosum* root and leaf

**DOI:** 10.3389/fchem.2024.1458505

**Published:** 2024-09-13

**Authors:** P. Kavya, R. C. Theijeswini, M. Gayathri

**Affiliations:** ^1^ Department of Bio Medical Sciences, School of Bio Sciences and Technology, Vellore Institute of Technology, Vellore, Tamil Nadu, India; ^2^ Department of Biotechnology, School of Bio Sciences and Technology, Vellore Institute of Technology, Vellore, Tamil Nadu, India

**Keywords:** *Chlorophytum comosum*, hypoglycemic, antioxidant, phytochemical, GC-MS, molecular docking

## Abstract

*Chlorophytum comosum* is a plant with medicinal potential traditionally used to treat different diseases. The present study aimed to determine the bioactive compounds, hypoglycemic and antioxidant potential of *C. comosum* root and leaf. The ethyl acetate extracts of *C. comosum* root and leaf were analyzed by GC-MS to determine the bioactive compounds. The hypoglycemic potential of the extracts was evaluated by α-amylase, α-glucosidase, glucose diffusion inhibitory assays, and glucose adsorption assay. The ethyl acetate extract of *C. comosum* root inhibited α-amylase, α-glucosidase, and glucose diffusion in a concentration-dependent manner with IC_50_ values of 205.39 ± 0.15, 179.34 ± 0.3 and 535.248 μg/mL, respectively, and the leaf extract inhibited α-amylase and α-glucosidase enzymes with IC_50_ values of 547.99 ± 0.09, and 198.18 ± 0.25 μg/mL respectively. *C. comosum* root and leaf extracts also improved glucose adsorption. Heptadecanoic acid and dodecanoic acid were identified as potential compounds with hypoglycemic properties through molecular docking. The extracts were also assessed for their antioxidant activity using DPPH, ABTS, and FRAP assays. *C. comosum* root and leaf extracts were also able to scavenge DPPH radicals with IC_50_ values of 108.37 ± 0.06 and 181.79 ± 0.09 µM and ABTS radicals with IC_50_ values of 126.24 ± 0.13 and 264.409 ± 0.08 µM, respectively. The root and leaf extracts also reduced the ferricyanide complex to ferrocyanide with higher reducing powers of 2.24 ± 0.02 and 1.65 ± 0.03, respectively. The results showed that the ethyl acetate extract of *C. comosum* root has significant antioxidant and hypoglycemic potential compared to the leaf extract. Thus, it can also be studied to isolate the potential compounds with antihyperglycemic activities.

## Graphical Abstract

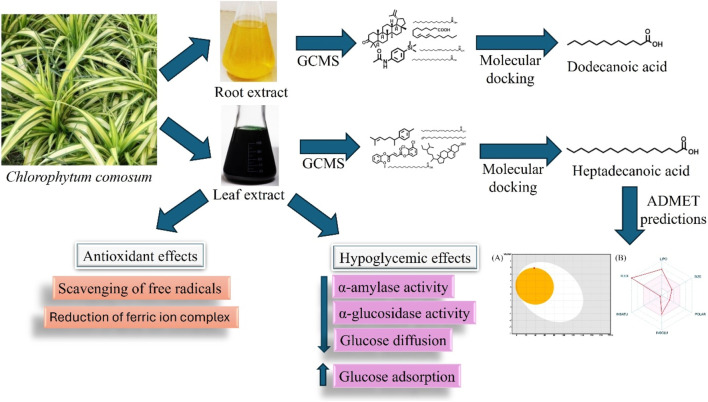

## Introduction

*Chlorophytum comosum* (*C. comosum*) is a species of the genus *Chrolorphytum* (Asparagaceae) and is distributed mainly in Africa, India, and Australia (Patil et al., 2015). The genus *Chrolorphytum* consists of more than 200 species, and a majority of the species belonging to this particular genus are called ‘medicinal herbs’ in India, Africa, China, Mongolia, and Tibet (Tabuti et al., 2003; Katoch et al., 2010; Deore et al., 2015; Fan et al., 2017). *C. comosum* pertains to an evergreen, perennial species of flowering plants indigenous to southern and tropical parts of Africa (Peng et al., 2020). It can grow around 60 cm in length on an approximate basis, even though it may descend multiple feet owing to being a hanging plant (Idrissi Azami et al., 2022). *C. comosum* is commonly referred to as spider plant and is more often ornamental, prominent houseplants (Li et al., 2021). Spider plants are widely known for their curving, tapering leaves, which possess distinct stripes of white and green, along with their characteristic prolonged, hooking stems with tiny plantlets, attributing to a unique appearance (Peng et al., 2020; Hubai et al., 2023). In conventional medicine, *C*. *comosum* has been used in China to treat asthma, bronchitis, burns, and fractures (Katoch et al., 2010). Due to the extended use of the *Chrolophytum* genus in conventional medicine, it has caught the interest of researchers globally in evidential medicine (Rzhepakovsky et al., 2022). Different classes of phytochemical compounds, including phenolics, alkaloids, saponins, flavonoids, steroids, terpenoids, carbohydrates, proteins, and tannins, have been identified in the *Chrolophytum* genus (Katoch et al., 2010; Khanam et al., 2013; Manokari et al., 2021). *C*. *comosum* leaf is reported to contain phenolic compounds, flavonoids, carotenoids, chlorophylls, reducing sugars, and tannins (Rzhepakovsky et al., 2022). It has also been reported to have anti-inflammatory, hepatoprotective, cardioprotective, antioxidant, neuroprotective, antimicrobial, antiproliferative, and immunomodulatory effects (Matsushita et al., 2005; Katoch et al., 2010; Basu and Jha, 2011; Shaker et al., 2021; Rzhepakovsky et al., 2022; Yagubova et al., 2023).

Type 2 Diabetes Mellitus (T2DM) is a chronic metabolic disorder with a highly abstruse pathogenesis (Himanshu et al., 2020). It is characterised by hyperglycemia or surging blood glucose levels due to anomalies in insulin production or insulin action (Banday et al., 2020). Genetic and environmental variables can contribute to the development of T2DM (Andrade-Cetto et al., 2021; Sharma et al., 2021). Certain ecological factors, such as calorie intake, air pollution, and physical inactivity, contribute significantly to the disease’s increasing prevalence (Wang et al., 2021). Hyperglycemia occurs in numerous forms, eventually leading to fat, protein, and carbohydrate metabolic dysfunctions (Giri et al., 2018). Prolonged periods of hyperglycaemic conditions often lead to both macrovascular and microvascular complications, which are considered prime factors in diabetes-related mortality cum morbidity. Chronic hyperglycaemic condition is also associated with neuropathy, retinopathy, cardiovascular dysfunction, and nephropathy. Therefore, all these compiled elevates the mortality cum morbidity statistics for diabetes patients worldwide (Galicia-Garcia et al., 2020). Even though the current anti-diabetic medicines are effective in glycemic control, they are often accompanied by harmful side effects (Khursheed et al., 2019). Most recurring adverse effects include low blood sugar or hypoglycaemia, obesity, gastrointestinal dysfunctions, and occasionally cardiovascular complications (Giri et al., 2018). Hence, developing medications with a lower risk profile and minimal side effects is essential (Pereira and Valado, 2023).

All living organisms undergo oxidative processes, which are essential metabolic processes. However, free radicals generated during the chain reactions of the oxidation process cause damage to cells (Di Meo and Venditti, 2020; Shlapakova et al., 2020). Antioxidants are extensively employed to inhibit or eliminate oxidative damage in the human body (Gulcin, 2020). Antioxidants neutralize free radicals by donating electrons (Al-Salahi et al., 2019). It has been demonstrated that antioxidant activity is essential for treating conditions such as diabetes, cancer, hypertension, cardiovascular disease, and dyslipidemia (Mao et al., 2023). Hence, natural products with antioxidant activity may be an alternative therapeutic option for managing these conditions (El-Shibani et al., 2023; Pham et al., 2023).

Hence, a rising interest has been in developing novel herbal medicines to treat hyperglycemia and oxidative stress (Cagliostro et al., 2022; Pereira and Valado, 2023). Herbal medicines are often observed to harness the efficacy of natural compounds with slighter side effects, providing a complementary or an alternative strategy towards oxidative stress and glycaemic control (Vivó-Barrachina et al., 2022). Many herbal plants possess phenolic compounds, flavonoids, terpenoids, glycosides, carotenoids, and alkaloids, known to have hypoglycemic and antioxidant properties (Pang et al., 2019). No scientific report shows that crude extracts of *C*. *comosum* root and leaves have hypoglycemic properties. Therefore, the present study analysed the phytochemical composition of different solvent extracts of *C. comosum* root and leaf and assessed the hypoglycemic and antioxidant potential of the ethyl acetate extracts *in vitro* and *in silico*. In addition, absorption, distribution, metabolism, excretion, and toxicity (ADMET) analysis was performed to predict the lead compound’s potential role as an oral drug.

## Materials and methods

### Chemicals and reagents

The required enzymes and chemicals, such as α-glucosidase, para-nitrophenyl glucopyranoside, dinitro salicylic acid, α-amylase, potato starch, glucose, dialysis membrane, Glucose oxidase-peroxidase (GOD-POD) kit, 2,2-diphenyl-1-picrylhydrazyl (DPPH), and 2,2′-azinobis (3-ethylbenzothiazoline-6-sulfonic acid (ABTS) were obtained from the Hi-Media Laboratories in Mumbai, India.

### Plant collection and authentication

The roots and leaves of the plant *C. comosum* were collected from the district of Vellore, Tamil Nadu, India. The plant was botanically identified and authenticated by Siddha Medicinal Plants Garden experts under the Ministry of AYUSH, Mettur, Tamil Nadu, Government of India (Accession No. C161122048C).

### Preparation of the plant extracts

The roots and leaves of *C. comosum* were thoroughly washed with distilled water and subsequently dried under shade at room temperature and coarsely ground to a powder with a functional mechanical grinder. The plant powder was extracted using the maceration procedure using five different solvents: hexane, ethyl acetate, ethanol, methanol, and water. 100 g of the plant powder was mixed with 1,000 mL of the solvents separately for 12 h at 600 rpm in a magnetic stirrer. The prepared extracts were then filtered using a Whatman filter paper No. 1 and concentrated at 45°C with a reduced pressure using a rotary evaporator (Tarsons Rockyvac, 300). Further, the sticky concentrate was lyophilised with subsequent storage at 4°C. The following equation was used to calculate the percentage of yield of different solvent extracts of *C. comosum* roots and leaves:

$$\%\;Yield=\frac{Weight\;of\;dried\;extract}{Weight\;of\;dried\;plant\;material}\times 100$$

### Qualitative phytochemical analysis

The presence of carbohydrates, proteins, reducing sugars, saponins, anthraquinones, phenolics, tannins, flavonoids, terpenoids, glycosides, anthocyanins, resins, fixed oils, and fat, quinones, lignins, sterols, alkaloids and anthraquinones in all the extracts were determined through qualitative phytochemical analysis using standard procedures (Oscar et al., 2020).

### Quantitative phytochemical analysis

#### Estimation of total phenolic content

The extract’s total phenolic content (TPC) was assessed using the Folin-Ciocalteu (FC) method, with certain modifications. Concisely, 100 µL of 1 mg/mL extracts and the standard gallic acid of 20, 40, 60, 80, 100, and 120 μg/mL concentrations were diluted to 4.6 mL with dimethyl sulfoxide (DMSO) and mixed with 100 µL of the FC reagent. Following a 3-min incubation period, the mixture was mixed with 300 µL of 2% Na_2_CO_3_. After incubating at 25°C for 90 min, the absorbance was read at 760 nm. TPC was represented as mg gallic acid equivalent/g of sample (mgGAE/g sample) (Aoussar et al., 2020).

#### Estimation of total flavonoid content

Determining total flavonoid content (TFC) in the extracts followed established procedures. Specifically, 500 μL of the 1 mg/mL extracts and quercetin of 20, 40, 60, 80, 100, and 120 μg/mL concentrations were prepared in DMSO and added with 75 μL of 5% NaNO_2_ and 150 μL of 10% AlCl_3_. After incubating for 5 min at 25°C, the mixture was added with 500 μL of 1 M NaOH reagent, and subsequently, the absorbance was measured at 510 nm. TFC was expressed as mg quercetin equivalent/g of sample (mgQUE/g sample) (Žilić et al., 2011).

#### FTIR analysis

The characteristic peaks and functional groups of the crude extracts (1 mg/mL; dissolved in analytical grade ethyl acetate) were identified through fourier transform infrared spectroscopy (Nicolet iS50, Thermo Scientific, United States) at a resolution of 4 cm^−1^ and a frequency range of 4,000–500 cm^−1^. The recorded FTIR peak values were used for analysis (Popescu et al., 2022).

#### Identification of compounds by GC-MS technique

Gas chromatography-mass spectrometry (GC-MS) analysis of ethyl acetate extracts of the roots and leaves of *C. comosum* was carried out to identify the compounds using the GC-MS technique (PerkinElmer Clarus 680/600). A capillary column setup (20 m in length × 0.18 mm in diameter, 1.00 µm in thickness of film) was used to separate the compounds. Helium was employed at a steady rate of 36.3 cm/sec as a carrier gas. The concentrated extract was dissolved in analytical-grade ethyl acetate at 1 mg/mL concentration and filtered using a 0.45 µm syringe filter. 2 μL of the extract was injected via an auto-injector into GC mod coupled with MS mod. The GC oven temperature was transitioned from 200°C to 150°C at 4°C per minute, maintaining the temperature constant for 5 min. The ion source’s temperature in the MS and the interface temperature remained constant at 230°C and 280°C, respectively. TurboMass software was used to collect the data, and the National Institute of Standards and Technology (NIST) Library 2005 was used to identify the compounds (Ms and Jayaraj, 2023).

#### Molecular docking

The 3-dimensional crystal configurations of α-amylase and α-glucosidase enzymes were downloaded from the protein data bank (PDB), specifically α-amylase (1hny) and α-glucosidase (3wy1). Utilising the swiss PDB viewer, the protein structures were prepared. The 3-dimensional structures of all ligands were downloaded from the PubChem repository of compounds and subsequently processed employing the Avogadro tool. Grid boxes were set to the dimensions of x = 17.03, y = 23.33, and z = 15.26 for α-amylase and x = 20.94, y = 23.99, and z = 25 for α-glucosidase at the geometric centre of the respective receptors to cover the active site amino acids. Preparation of the grid boxes and docking procedures were carried out utilising autodock vina via the virtual screening tool PyRx. The binding energy of each of the ligands was computed, and the interaction between protein and ligand was visualised with PyMOL and biovia discovery studio (Quimque et al., 2022).

#### ADME analysis

The isomeric SMILES of all the compounds were downloaded from the PubChem repository of compounds (http://pubchem.ncbi.nlm.nih.gov/), and their ADME characteristics were assessed using the swissADME tool (http://www.swissadme.ch/) (Alam et al., 2023). The toxicity of all the compounds was assessed using the ADMET Lab server (https://admet.scbdd.com/) (Saeed et al., 2022).

### Evaluation of antihyperglycemic activity

#### α-amylase assay

Six varying concentrations (100, 200, 300, 400, 500, 600 μg/mL) of the extracts and the standard drug acarbose were prepared in DMSO. 1 mL of the extracts and acarbose was added with 1 mL of 0.5 mg/mL α-amylase dissolved in sodium phosphate buffer of 0.02 M, pH 6.9 with 0.006 M NaCl, and then incubated at 25°C for 10 min. Subsequently, 500 *μ*L of 1% potato starch solution (substrate) was mixed with it, and the mixture was incubated at 25°C for 10 min. The reaction was terminated by mixing with 1 mL of 1% di nitro salicylic acid reagent. After incubating in a water bath at 100°C for 5 min, the mixture was cooled to room temperature. Then, the reaction mixture was diluted to 10 mL with sodium phosphate buffer of 0.02 M, pH 6.9 with 0.006 M NaCl. Absorbance was measured at 540 nm (Roy and Mahalingam, 2017). The experiment was conducted in triplicate. The α-amylase inhibition percentage can be computed using the subsequent formula:

$$\%\text{ of inhibition of }\text{α}\text{‐amylase}=\frac{\text{Abs}.\;\text{of}\;\text{control}\;-\;\text{Abs}.\;\text{of}\;\text{sample}}{\text{Abs}.\;\text{of}\;\text{control}}\times 100$$

#### α-glucosidase assay

Six varying concentrations (100, 200, 300, 400, 500, 600 μg/mL) of extracts and the standard drug acarbose were prepared in DMSO. 1 mL of the extracts and acarbose was mixed with an equal amount of 0.1 unit α-glucosidase. The reaction was initiated by mixing with 500 *μ*L of 3 mM p-nitrophenyl glucopyranoside (substrate). The reaction proceeded at 37°C for 25 min. The reaction was terminated by mixing with 1 mL of 0.02 M Na_2_CO_3_, and the mixture was incubated at 25°C for 10 min. Subsequently, the reaction mixture was diluted to 10 mL with 0.02 M sodium phosphate buffer of pH 6.9 with 0.006 M NaCl. By measuring the release of p-nitrophenol from p-nitrophenyl glucopyranoside, the inhibitory activity of α-glucosidase was assessed at 405 nm (Roy and Mahalingam, 2017). The α-glucosidase inhibition percentage can be computed using the subsequent formula:

$$\%\text{ of inhibition of }\alpha \text{‐glucosidase}=\frac{\text{Abs}.\;\text{of}\;\text{control}\;-\;\text{Abs}.\;\text{of}\;\text{sample}}{\text{Abs}.\;\text{of}\;\text{control}}\times 100$$

#### Glucose diffusion inhibitory assay

Six varying concentrations (100, 200, 300, 400, 500, 600 *μ*g/mL) of extracts and the standard drug, acarbose were prepared in DMSO. 1 mL of the extract and acarbose was placed in an 8 cm long dialysis membrane strip (12,000 MW) along with 1 mL of 0.22 mM glucose solution prepared in 0.15 M NaCl. The dialysis membrane was then placed in a mixture of 40 mL of NaCl solution (0.15 M) and 10 mL of distilled water after being sealed on both ends. Control contained 1 mL of 0.22 mM glucose solution prepared in 0.15 M NaCl and 1 mL of distilled water. The mixture was then allowed to mix in an orbital shaker at 150 rpm for 3 h. Every 30 min, the glucose content of the mixture was quantified at 540 nm using the DNSA method. The experiment was conducted in triplicate (Roy and Mahalingam, 2017). The glucose diffusion inhibition percentage can be computed by using the subsequent formula:

$$\%\text{ of inhibition of glucose diffusion}=\frac{\text{Abs}.\;\text{of}\;\text{control}\;-\;\text{Abs}.\;\text{of}\;\text{sample}}{\text{Abs}.\;\text{of}\;\text{control}}\times 100$$

#### Glucose adsorption assay

Six varying concentrations (100, 200, 300, 400, 500, 600 *μ*g/mL) of extracts and the standard drug acarbose were prepared in DMSO. 1 mL of each concentration was mixed with 1 mL of glucose in 5, 10, and 25 mM concentrations. After thoroughly mixing, the mixtures were then incubated in a shaker water bath at 37 C for 6 h. After incubation, the mixtures were subjected to centrifugation at 4,000 rpm for 15 min, and the supernatant was collected. The GOD-POD kit method was used to measure the glucose content in the supernatant (Rehman et al., 2018). The bound glucose was quantified using the subsequent formula:

$$\text{Glucose bound}=\frac{G1\;-\;G6}{\text{weight}\;\text{of}\;\text{sample}}\times \text{volume of sample}$$

where *G*1 represents the initial glucose concentration, and *G*6 represents the glucose concentration after 6 h.

### Evaluation of antioxidant activity

#### DPPH radical scavenging assay

DPPH shows absorption at 517 nm in its radical state. However, when it undergoes reduction due to an antioxidant or a radical species, its absorption decreases. Concisely, six different concentrations (100, 200, 300, 400, 500, 600 μg/mL) of extracts and the standard (ascorbic acid) were prepared in DMSO. 1 mL of the extracts and ascorbic acid was mixed with 1 mL of the 25 μg/mL DPPH solution in ethanol. The mixture was incubated at 25°C for 40 min in the dark. Then, the absorbance was measured at 517 nm (Fu et al., 2021). Decreased absorbance of the reaction mixture suggests higher free radical scavenging activity. The subsequent equation was used to calculate the DPPH radical scavenging activity:

$$\text{Scavenging rate }\%=\left(1-\frac{A_{1}-\;A_{2}}{A_{0}}\right)\times 100\%$$

A_0_ = Absorbance of negative control.A_1_ = Absorbance of DPPH with sample.A_2_ = Absorbance of sample background.

#### ABTS radical scavenging assay

In this assay, the antioxidant activity was assessed by measuring the reduction of blue-green ABTS radical. Concisely, ABTS (7 mM/L) with potassium persulfate (2.45 mM/L) was dissolved in 5 mL of distilled water. The solution was incubated at 25°C in dark condition for 12–16 h. Then the ABTS solution was diluted with ethanol (1:89 v/v). 2 mL of the formed ABTS solution was added with 20 µL of extracts and standard (ascorbic acid) of different concentrations (100, 200, 300, 400, 500, 600 μg/mL) dissolved in DMSO. The mixture was incubated at 25°C in a dark condition for 30 min. The absorbance was read at 734 nm (Senthilkumar et al., 2013). The subsequent equation was used to calculate the ABTS radical scavenging activity:

$$\text{Scavenging rate }\%=\left(1-\frac{A_{1}-\;A_{2}}{A_{0}}\right)\times 100\%$$

A_0_ = Absorbance of negative control.A_1_ = Absorbance of ABTS with sample.A_2_ = Absorbance of sample background.

#### Reducing power assay

This assay relies on reducing Fe^3+^ ions in K_3_ [Fe(CN)_6_]to Fe^2+^ ions in the presence of antioxidants under acidic conditions. A higher absorbance value indicates a higher reduction potential. Concisely, 1 mL of the extracts and standard (ascorbic acid) of different concentrations (100, 200, 300, 400, 500, 600 μg/mL) prepared in DMSO was added with 2.5 mL of phosphate buffer (0.2 mol/L, pH 6.6) and 2.5 mL of 1% K_3_ [Fe(CN)_6_]. The mixture was incubated in the water bath at 50°C for 20 min. After the mixture was cooled to room temperature, 2.5 mL of 10% CCl_3_COOH was added. Then, the mixture was centrifuged at 6,000 rpm for 10 min at room temperature. 2.5 mL of supernatant was collected and added with distilled water (2.5 mL) and 0.1% FeCl_3_ (0.5 mL). The reaction mixture was incubated for 10 min at 25°C. The absorbance was read at 700 nm (Fu et al., 2021).

#### Statistical analysis

Data was represented as mean ± SD. One-way analysis of variance and JMP Pro 17 were employed for the statistical analysis. *p* values less than 0.05 were deemed statistically significant.

## Results and discussion

### Percentage of the yield of various solvent extracts of *C. comosum* root and leaf

The percentage of yield of various solvent extracts of *C. comosum* root and leaf is given in Table 1. Among the five extracts, water extract exhibited a high percentage of yield (7.88% ± 0.33% and 16.81% ± 0.46%), and hexane extract exhibited a low percentage of yield (0.29% ± 0.23% and 0.93% ± 0.42%) for roots and leaves extracts of *C. comosum* respectively. Ethyl acetate, ethanol, and methanol extracts of *C. comosum* roots had 0.99 ± 0.35, 1.82% ± 0.26% and 4.25% ± 0.44% of yield and ethyl acetate, ethanol, and methanol extracts of *C. comosum* leaves had 2.53 ± 0.32, 1.72% ± 0.27% and 4.84% ± 0.37% of yield respectively.

**TABLE 1:** Percentage of the yield of various solvent extracts of *C. comosum* roots and leaves.

*Chlorophytum comosum*	Root extract yield (%)	Leaf extract yield (%)
Hexane extract	0.29 ± 0.23	0.93 ± 0.42
Ethyl acetate extract	0.99 ± 0.35	2.53 ± 0.32
Ethanol extract	1.82 ± 0.26	1.72 ± 0.27
Methanol extract	4.25 ± 0.44	4.84 ± 0.37
Water extract	7.88 ± 0.33	16.81 ± 0.46

Note: The values are represented as the standard deviation of the mean (means ± SD), n = 3.

### Qualitative phytochemical profile

The qualitative analysis of phytochemicals present in different solvent extracts of *C. comosum* root and leaf is represented in tables (Tables 2, 3). Both roots and leaf extracts of *C. comosum* exhibit a rich array of phytochemicals, including flavonoids, phenolic compounds, proteins, tannins, glycosides, carbohydrates, and quinones. Cardiac glycosides, anthocyanins, anthraquinones, leucoanthocyanins, terpenoids, and fixed oils and fats were absent in both roots and leaves extracts of *C. comosum*. Saponins were present in the roots extract but absent in the leaves extract of *C. comosum*. Ethanolic and methanolic root extracts of *C. comosum* show similar phytochemical profiles and possess more phytochemical compounds, and hexane extract possesses fewer phytochemical compounds than other solvent extracts. Leaf extracts exhibit fewer phytochemicals than the root extracts of *C. comosum*. Phenolic compounds, flavonoids, proteins, glycosides, carbohydrates, tannins, alkaloids, phytosterols, quinones, and were present, and cardiac glycosides, terpenoids, anthocyanins, saponins, fixed oils and fats, anthraquinones, phlorotannins, leucoanthocyanins, and lignins were absent in leaf extracts of *C. comosum*. Phenolic compounds, flavonoids, alkaloids, and tannins are significant phytochemicals and have been previously documented in many other species of *Chlorophytum* (Khanam et al., 2013; Mechchate et al., 2021). The methanol, hexane, chloroform, n-butanol, and water extracts of *C*. *comosum* leaf contain phenolic compounds, flavonoids, chlorophylls, carotenoids, tannins, and reducing sugars (Rzhepakovsky et al., 2022). Phytochemical compounds detected in different solvent extracts of *C*. *comosum* root and leaf have been reported to have therapeutic properties beneficial for conditions such as hyperglycemia, hyperlipidemia, inflammation, coronary artery disease, and cancer (Matsushita et al., 2005; Rzhepakovsky et al., 2022)

**TABLE 2:** Qualitative phytochemical analysis of various solvent extracts of *C. comosum* root.

Phytoconstituent	Hexane	Ethyl acetate	Ethanol	Methanol	Water
Carbohydrates	–	–	+	+	+
Glycosides	–	+	+	+	+
Cardiac glycosides	–	–	–	–	–
Proteins and amino acids	+	+	+	+	+
Alkaloids	+	+	+	+	+
Flavonoids	+	+	+	+	+
Phenolic compounds	+	+	+	+	+
Tannins	+	+	+	+	+
Phlobatannins	+	+	+	+	+
Saponins	–	–	+	+	–
Phytosterols	+	+	+	+	–
Terpenoids	–	–	–	–	–
Lignins	–	–	–	–	–
Quinones	+	+	+	+	–
Anthraquinones	–	–	–	–	–
Anthocyanins	–	–	–	–	–
Leucoanthocyanins	–	–	–	–	–
Fixed oils and fat	–	–	–	–	–

+ presence and–absence.

**TABLE 3:** Qualitative phytochemical analysis of various solvent extracts of *C. comosum* leaf.

Phytoconstituent	Hexane	Ethyl acetate	Ethanol	Methanol	Water
Carbohydrates	+	+	–	+	+
Glycosides	+	+	+	+	+
Cardiac glycosides	–	–	–	–	–
Proteins and amino acids	+	+	-	+	+
Alkaloids	+	–	–	–	–
Flavonoids	+	+	+	+	+
Phenolic compounds	+	+	+	+	+
Tannins	+	+	+	+	+
Phlobatannins	–	–	–	–	–
Saponins	–	–	–	–	–
Phytosterols	–	+	–	–	+
Terpenoids	–	–	–	–	–
Lignins	–	–	–	–	–
Quinones	+	+	+	+	–
Anthraquinones	–	–	–	–	–
Anthocyanins	–	–	–	–	–
Leucoanthocyanins	–	–	–	–	–
Fixed oils and fat	–	–	–	–	–

+ presence and – absence.

### Quantitative phytochemical profile

#### Total phenolics and flavonoid content

**FIGURE 1:**
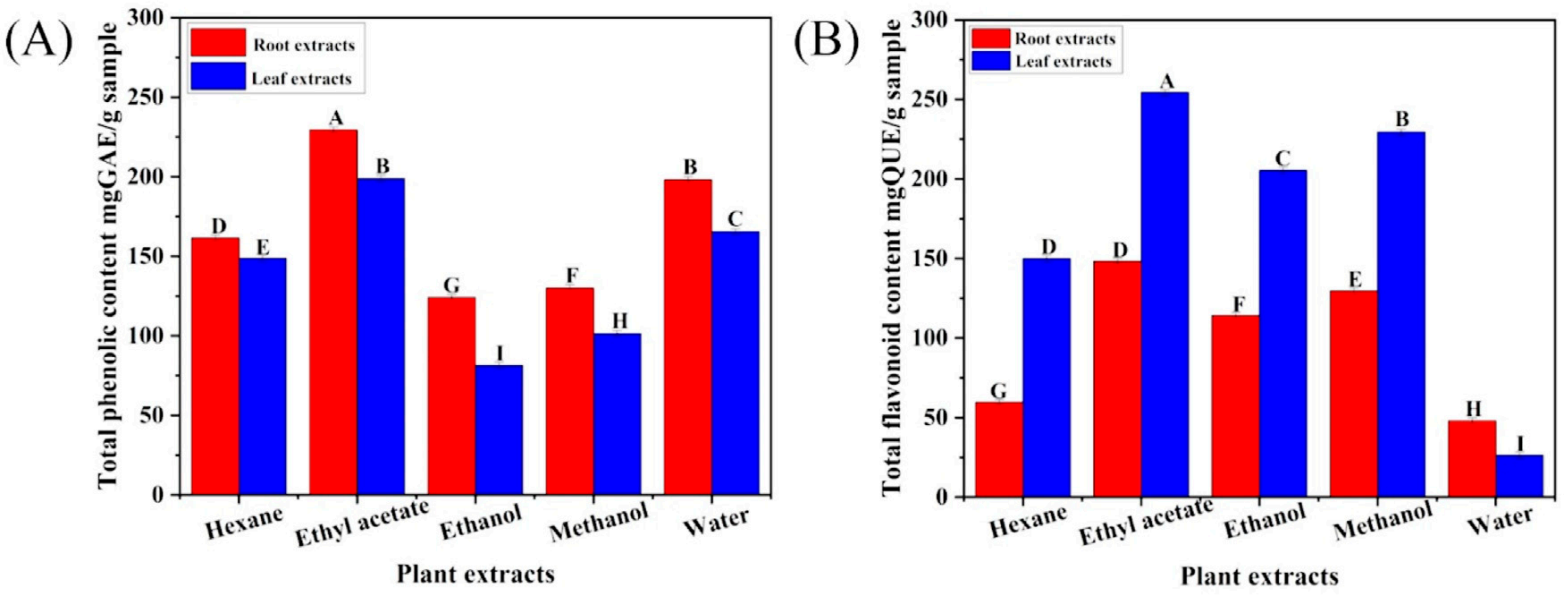
**(A)** Total phenolics concentration in different solvent extracts of *C. comosum* roots and leaves, **(B)** Total flavonoids concentration in different solvent extracts of *C. comosum* roots and leaves. Different letters show significant differences between the tested extracts (*p* < 0.05).

The total phenolic and flavonoid content of different solvent extracts of *C. comosum* root and leaf were represented as mgGAE/g sample and mgQUE/g sample, respectively. Both phenolic and flavonoid compounds were notably abundant in the plant material, although there were some variations among the extraction solvents (Figures 1A, B). The ethyl acetate extracts of *C. comosum* root and leaf had high phenolic content (229.33 ± 1.38, and 198.66 ± 1.58 mgGAE/g sample) in comparison with hexane, ethanol, methanol, and water extracts which exhibited 161.33 ± 1.78, 124 ± 1.84, 130 ± 0.95, 198 ± 1.69, 148.66 ± 1.48, 81.33 ± 1.54, 101.33 ± 1.74, and 165.33 ± 1.65 mgGAE/g sample respectively. The ethyl acetate extracts of *C. comosum* root and leaf also had high flavonoid content (148.18 ± 0.95, and 254.24 ± 1.29 mgQUE/g sample) in comparison with hexane, ethanol, methanol, and water extracts which exhibited 59.54 ± 1.57, 114.09 ± 1.25, 129.54 ± 1.64, 47.88 ± 0.98, 149.85 ± 1.64, 205.3 ± 1.91, 229.39 ± 1.82, and 26.21 ± 0.98 mgQUE/g sample respectively. The plant extract’s large phenolics and flavonoid content contribute to promising health improvising properties. Flavonoids are naturally occurring compounds with diverse phenolic structures found in plants, along with phenolic compounds that are extensively found in plants and own various pharmacological properties such as hypoglycemic, hypolipidemic, anticancer, anti-inflammatory, antioxidant, cardioprotective effects, etc., (Singh et al., 2022). The ethyl acetate extracts of *C*. *comosum* root and leaf contained comparatively higher flavonoid and phenolic content, so they were chosen for further analyses and studies.

#### FTIR spectra of *C. comosum* root and leaf ethyl acetate extracts

**FIGURE 2:**
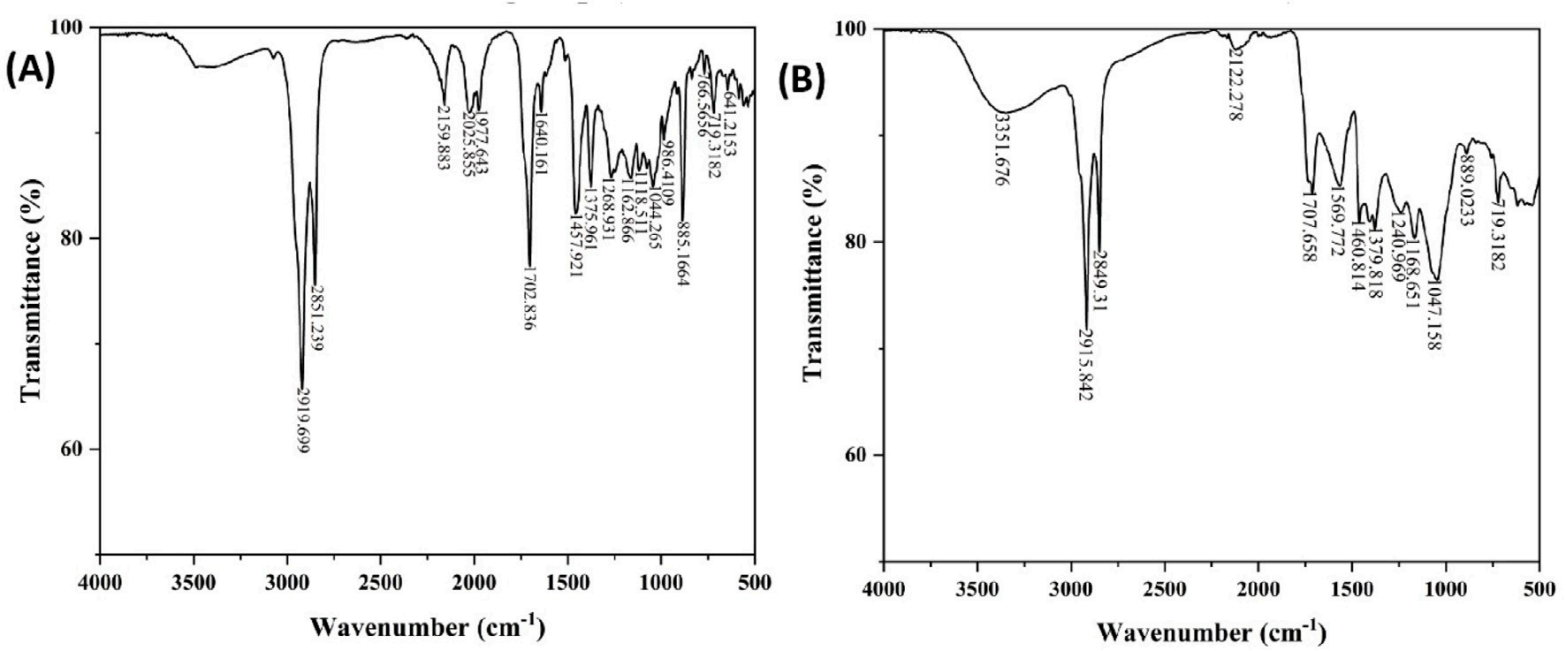
**(A)** FTIR spectra of ethyl acetate extract of *C. comosum* root, **(B)** FTIR spectra of ethyl acetate extract of *C. comosum* leaf.

The infrared spectrum of the ethyl acetate extracts of *C. comosum* root and leaf over the frequency range of 4,000–500 cm^−1^ was obtained to detect the functional groups present in the bioactive compounds by analysing the distinctive absorption peaks in the fingerprint region (Figures 2A, B).

The ethyl acetate extract of *C. comosum* root shows peaks at 2919.699 and 2851.239 cm^−1^, indicating stretching vibrations of the alkanes. The peaks at 2159.883 and 641.2153 cm^−1^ represent stretching and bending vibrations of the alkynes, respectively. The peak at 1702.836 cm^−1^ indicates the stretching of aldehydes, while the peak at 1,640.161 cm^−1^ represents the stretching vibrations of the C ≡ C group. The peak at 1,457.921 cm^−1^ suggests the bending vibrations of amines and amides, indicating the presence of amino acids or proteins. Furthermore, the peak at 1,375.961 cm^−1^ indicates the stretching of nitro compounds, and the peaks at 1,268.931 and 1,162.866 cm^−1^ represent the stretching of esters and ethers, respectively. The peaks at 1,118.511 and 1,044.265 cm^−1^ suggest the stretching of alcohols. Finally, the peaks at 986.4109, 885.1664, and 719.3182 cm^−1^ are suggestive of the bending vibrations of alkenes, while the peaks at 766.5656 and 641.2153 cm^−1^ suggest alkyl halides and alkynes stretching and bending vibrations.

The ethyl acetate extract of *C. comosum* leaf shows one characteristic peak at 3351.676 cm^−1^, representing the O-H group’s stretching vibration. This may indicate the presence of phenolics or phytosterols. Stretching vibrations of the C-H group were detected at 2915.842 and 2849.31 cm^−1^. The C ≡ C group’s stretching vibrations were detected at 2122.278 cm^−1^. The stretching of aldehydes was indicated at 1707.658 cm^−1^. The stretching of nitro compounds was detected at 1,569.772 cm^−1^. The peak at 1,460.814 cm^−1^ represented the bending vibrations of amines and amides, suggesting the presence of amino acids or proteins. The peaks at 1,379.818 and 889.0233 cm^−1^ indicated the bending of alkenes. The stretching of alkyl halides was recommended by the peak at 1,168.651 cm^−1^. The stretching of alcohols was represented by the peak at 1,047.158 cm^−1^. The peak at 719.3182 cm^−1^ represented the bending vibrations of aromatic rings.

The unique peaks of different functional groups confirmed the presence of phenols, flavonoids, aldehydes, and proteins. The ameliorative properties of antioxidants, antidiabetics, etc., are attributed to the O-H group (Badmus et al., 2020; Azeem et al., 2023).

#### GCMS profile of *C. comosum* root and leaf ethyl acetate extracts

**FIGURE 3:**
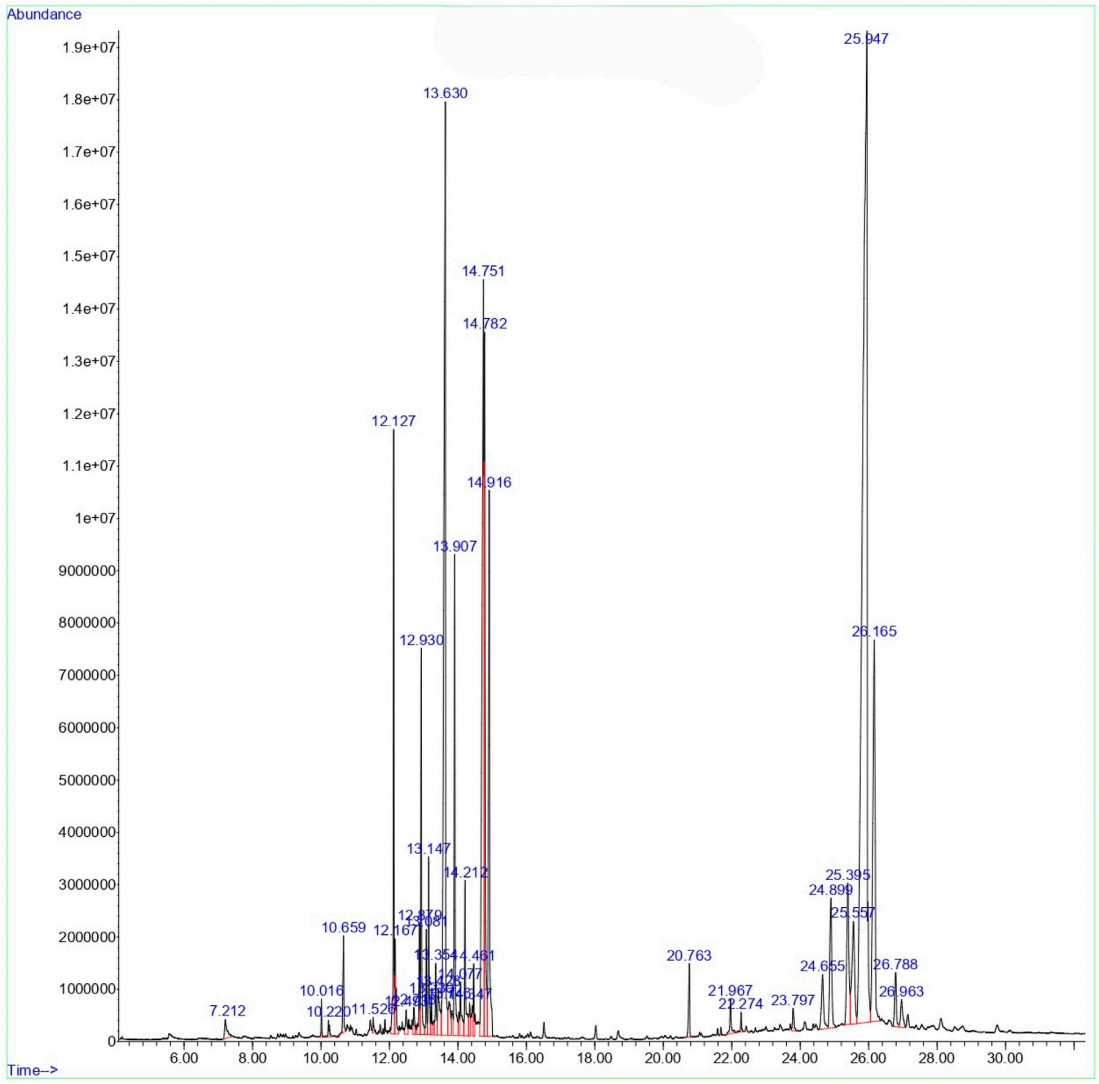
GCMS chromatogram of ethyl acetate extract of *C. comosum* root.

**FIGURE 4:**
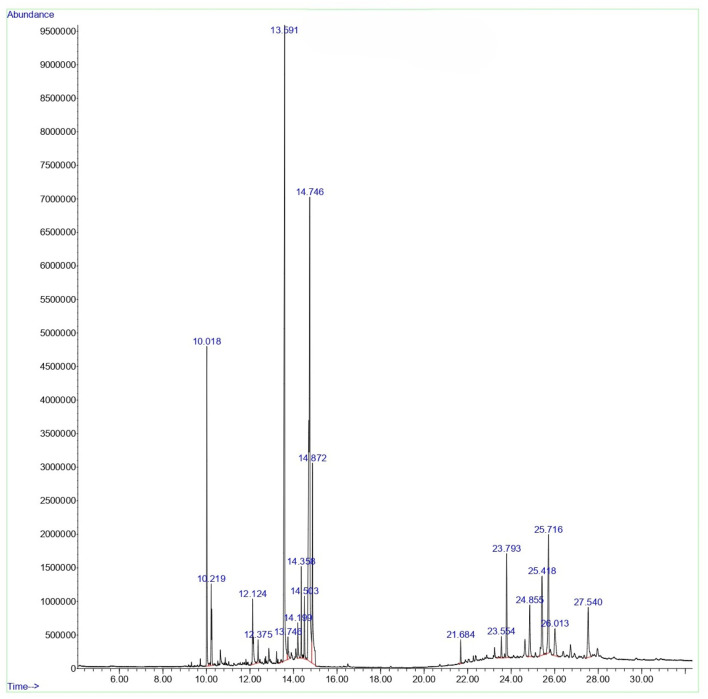
GCMS chromatogram of ethyl acetate extract of *C. comosum* leaf.

The compounds identified in *C*. *comosum* root and leaf ethyl acetate extracts are listed according to their column elution time (Tables 4, 5). GCMS chromatograms of the extracts are depicted in Figures 3, 4. GCMS analysis of the ethyl acetate extracts of *C. comosum* root and leaf detected 38 and 19 compounds, respectively. The major compounds detected in the root extract are lup-20 (29)-en-3-one, n-hexadecanoic acid, 9(E),11(E)-conjugated linoleic acid, acetamide,N-[4-(trimethylsilyl)phenyl]-, oleic Acid, and octadecanoic acid which constituted 33.49, 11.50, 10.32, 6.40, 5.23, and 4.75% respectively. The major compounds detected in the leaf extract are 9,12,15-octadecatrienoic acid, (Z,Z,Z)-, n-hexadecanoic acid, octadecanoic acid, benzene, 1-(1,5-dimethyl-4-hexenyl)-4-methyl-, fumaric acid, 2-methoxyphenyl 2,3-dichlorophenyl ester, and γ-sitosterol which constituted 27.80, 24.59, 7.53, 6.51, 5.65, and 4.25% respectively. Previous studies also reported n-hexadecanoic acid, 9,12,15-octadecatrienoic acid, (Z,Z,Z)-, and γ-sitosterol as major components of the *C*. *comosum* extracts (Rzhepakovsky et al., 2022).

**TABLE 4:** Compounds identified in ethyl acetate extract of *C. comosum* root.

Peak	Retention time	Molecular weight	Molecular formula	Name of compound	Structure of compound	Peak area	Peak area %
1	7.212	122.12	C₇H₆O₂	Benzoic acid	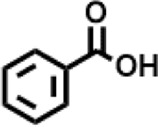	17,953,280	0.34
2	10.016	202.3352	C_15_H_22_	Benzene, 1-(1,5-dimethyl-4-hexenyl)-4-methyl-	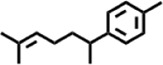	9,216,415	0.18
3	10.220	206.3239	C_14_H_22_O	2,4-Di-tert-butylphenol	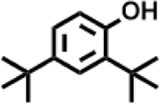	7,627,757	0.15
4	10.659	200.3178	C_12_H_24_O_2_	Dodecanoic acid	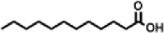	34,655,928	0.66
5	11.526	206.32	C_14_H_22_O	2,2-Dimethyl-2-[2.3,5,6-tetramethyl]ethanol	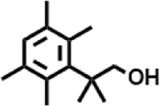	5,611,721	0.11
6	12.127	218.3346	C_15_H_22_O	Phenol, 5-(1,5-dimethyl-4-hexenyl)-2-methyl-, (R)-	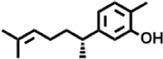	161,431,939	3.08
7	12.167	228.3709	C_14_H_28_O_2_	Tetradecanoic acid	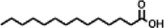	41,430,239	0.79
8	12.493	202.21	C_12_H_10_O_3_	Methyl 2-methoxy-1-naphthoate	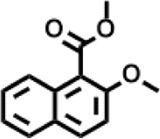	12,844,998	0.25
9	12.718	218.2054	C_12_H_10_O_4_	Ethanone, 1.1’-(6-hydroxy-2,5-benzofurandiyl)bis-	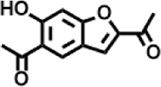	13,738,012	0.26
10	12.879	242.3975	C_15_H_30_O_2_	Pentadecanoic acid	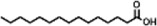	37,048,866	0.71
11	12.930	234.33	C_15_H_22_O_2_	3,5-Di-tert-butyl-2-hydroxybenzaldehyde	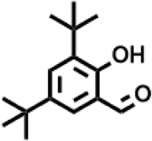	119,354,920	2.28
12	13.081	178.1846	C_10_H_10_O_3_	(E)-3-(2-Methoxyphenyl)-2-propenoic acid	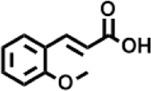	30,092,476	0.57
13	13.147	178.231	C_11_H_14_O_2_	Methyleugenol	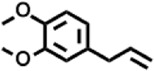	60,804,340	1.16
14	13.230	276.3707	C_17_H_24_O_3_	7,9-Di-tert-butyl-1-oxaspiro (4,5)deca-6,9-diene-2,8-dione	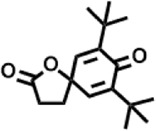	14,239,575	0.27
15	13.354	222.40	C_13_H_22_OSi	Benzyloxy (butyl)dimethylsilane	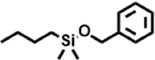	28,004,858	0.53
16	13.428	254.414	C_16_H_30_O_2_	Palmitoleic acid	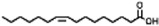	40,576,771	0.77
17	13.630	256.4241	C_16_H_32_O_2_	n-Hexadecanoic acid	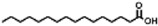	602,824,807	11.50
18	13.746	280.5316	C_20_H_40_	1-Eicosene	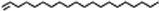	30,558,911	0.58
19	13.907	321.4	C_21_H_23_NO_2_	Benzoic acid, 4-heptyl-, 4-cyanophenyl ester	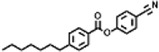	167,294,131	3.19
20	14.077	282.4614	C_18_H_32_O	cis-10-Heptadecenoic acid	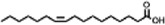	33,648,444	0.64
21	14.212	268.4	C_17_H_34_O_2_	Heptadecanoic acid	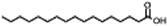	65,744,854	1.25
22	14.347	252.486	C_18_H_36_	1-Octadecene	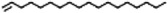	17,574,186	0.34
23	14.461	282.5044	C_19_H_38_O	2-Nonadecanone	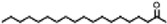	23,037,476	0.44
24	14.751	280.4	C_18_H_32_O_2_	9(E),11(E)-Conjugated linoleic acid	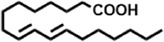	541,050,480	10.32
25	14.782	282.46	C_18_H_34_O_2_	Oleic Acid	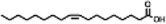	274,223,790	5.23
26	14.916	284.4772	C_18_H_36_O_2_	Octadecanoic acid	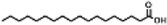	248,940,960	4.75
27	20.763	366.7070	C_26_H_54_	Hexacosane	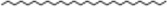	31,112,035	0.59
28	21.967	288.34	C_17_H_20_O_4_	1,1-Biphenyl, 2,3,4,4′-tetramethoxy-5-methyl-6′-methoxycarbonyl-	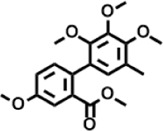	17,510,788	0.33
29	22.274	408.6	C_29_H_60_	Nonacosane	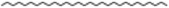	7,433,494	0.14
30	23.797	430.7	C_29_H_50_O_2_	dl-α-Tocopherol	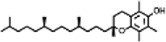	12,463,370	0.24
31	24.655	400.7	C_28_H_48_O	Campesterol	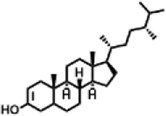	47,460,416	0.91
32	24.899	412.69	C_29_H_48_O	Stigmasterol	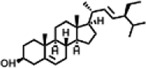	102,411,968	1.95
33	25.395	424.7	C_30_H_48_O	Ursa-9 (11),12-dien-3-ol	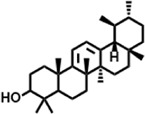	119,504,751	2.28
34	25.557	414.7067	C_29_H_50_O	γ -Sitosterol	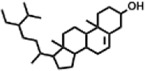	112,865,186	2.15
35	25.947	424.7015	C_30_H_48_O	Lup-20 (29)-en-3-one	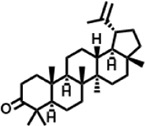	1,755,169,441	33.49
36	26.165	207.34	C_11_H_17_NOSi	Acetamide, N-[4-(trimethylsilyl)phenyl]-	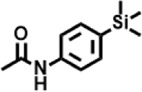	335,679,018	6.40
37	26.788	412.6908	C_29_H_48_O	Stigmast-4-en-3-one	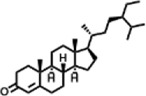	36,176,184	0.69
38	26.963	430.6	C_27_H_42_O_4_	Pennogenin	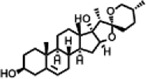	23,757,774	0.45

**TABLE 5:** Compounds identified in ethyl acetate extract of *C. comosum* leaf.

Peak	Retention time	Molecular weight	Molecular formula	Name of compound	Structure of compound	Peak area	Peak area %
**1**	10.018	202.3352	C_15_H_22_	Benzene, 1-(1,5-dimethyl-4-hexenyl)-4-methyl-	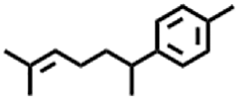	59,681,274	6.51
**2**	10.219	206.32	C_14_H_22_O	2,4-Di-tert-butylphenol	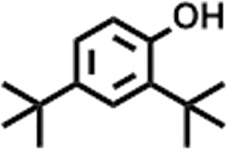	27,178,922	2.97
**3**	12.124	218.3346	C_15_H_22_O	Phenol, 5-(1,5-dimethyl-4-hexenyl)-2-methyl-, (R)-	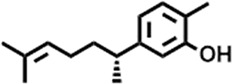	24,402,567	2.66
**4**	12.375	252.5	C_18_H_36_	1-Octadecene	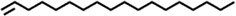	6,121,624	0.67
**5**	13.591	256.4241	C_16_H_32_O_2_	n-Hexadecanoic acid	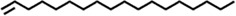	225,436,167	24.59
**6**	13.746	280.5316	C_20_H_40_	1-Eicosene	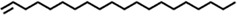	7,725,835	0.84
**7**	14.199	270.45	C_17_H_34_O_2_	Heptadecanoic acid	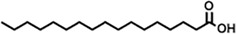	9,124,460	1.00
**8**	14.358	272.4681	C_20_H_32_	Kaur-16-ene	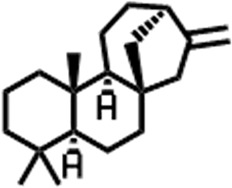	17,315,487	1.89
**9**	14.503	296.53	C_20_H_40_O	Phytol	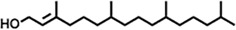	14,964,196	1.63
**10**	14.746	278.4296	C_18_H_30_O_2_	9,12,15-Octadecatrienoic acid, (Z,Z,Z)-	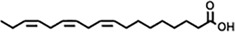	254,839,403	27.80
**11**	14.872	284.4772	C_18_H_36_O_2_	Octadecanoic acid	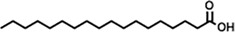	69,047,274	7.53
**12**	21.684	410.7	C_29_H_46_O	28-Norolean-17-en-3-one	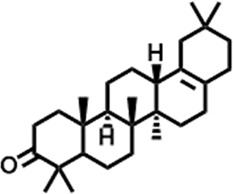	5,406,655	0.59
**13**	23.554	398.5766	C_23_H_42_O_5_	1,2-Cyclohexanedicarboxylic acid, dodecyl 2-ethoxyethyl ester	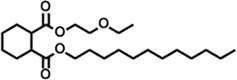	6,624,571	0.72
**14**	23.793	430.7061	C_29_H_50_O_2_	alpha-tocopherol	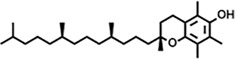	31,104,929	3.39
**15**	24.855	412.69	C_29_H_48_O	Stigmasterol	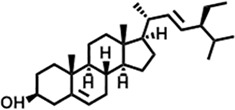	21,543,623	2.35
**16**	25.418	414.7067	C_29_H_50_O	γ -Sitosterol	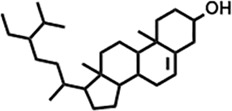	38,982,926	4.25
**17**	25.716	351.181	C_17_H_12_Cl_2_O_4_	Fumaric acid, 2-methoxyphenyl 2,3-dichlorophenyl ester	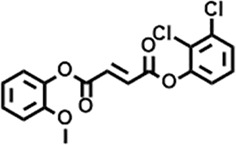	51,819,910	5.65
**18**	26.013	207.2704	C_15_H_13_N	Benzo [h]quinoline, 2,4-dimethyl-	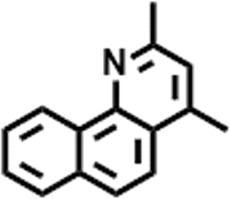	17,373,249	1.90
**19**	27.540	452.8	C_30_H_60_O_2_	Octacosanoic acid, ethyl ester	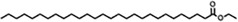	27,924,025	3.05

#### Molecular docking

**FIGURE 5:**
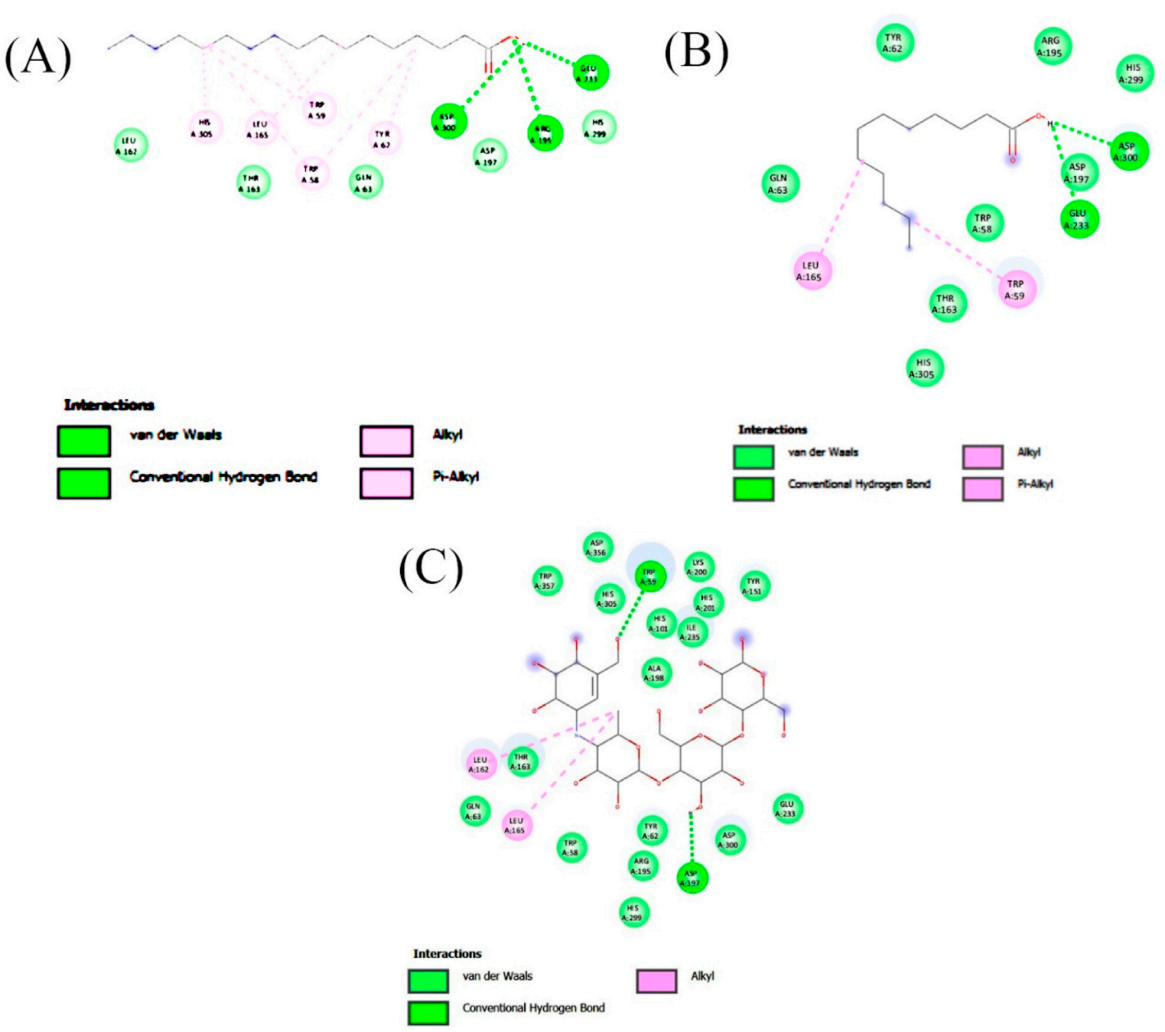
2-D interaction plot of the best-docked compounds into the active site of α-amylase **(A)** Heptadecanoic acid, **(B)** Dodecanoic acid, **(C)** Acarbose.

**FIGURE 6:**
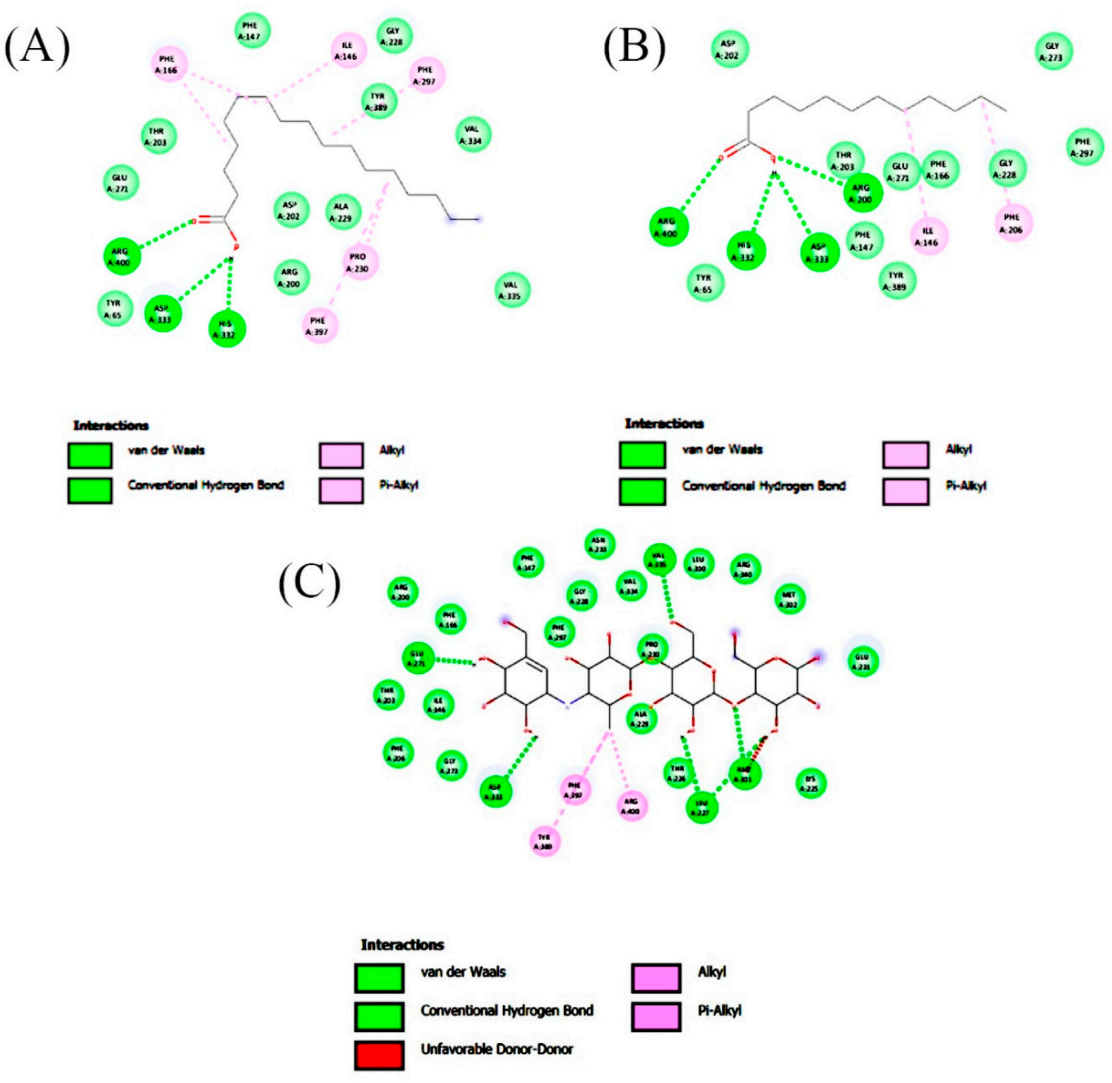
2-D interaction plot of the best-docked compounds into the active site of α-glucosidase **(A)** Heptadecanoic acid, **(B)** Dodecanoic acid, **(C)** Acarbose.

Molecular docking analysis was carried out to model the interactions of the compounds in the ethyl acetate extracts of *C. comosum* root and leaf with α-amylase and α-glucosidase enzymes. The analysis findings exhibit the potential affinities of the compounds with the enzymes. The binding affinities of all the compounds with α-amylase and α-glucosidase are given in Tables 6, 7, and the interactions of the most potent compounds with the receptors are shown in Figures 5, 6. The findings of the molecular docking analysis of compounds of ethyl acetate extract of *C*. *comosum* root with α-amylase exhibited that ursa-9 (11),12-dien-3-ol has the highest binding affinity of −10 kcal/mol with the enzyme, in comparison with other compounds. But ursa-9 (11),12-dien-3-ol formed a hydrogen bond with only one of the critical amino acid residues of the enzyme activity, ASP 197. Lup-20 (29)-en-3-one and stigmasterol also exhibited high binding affinity to the α-amylase enzyme, i.e., −9.6 and −9.4 kcal/mol, respectively. Still, these compounds didn’t establish conventional hydrogen bonds with the critical amino acid residues associated with the enzyme activity. The compounds dodecanoic acid and heptadecanoic acid bound with α-amylase with lower binding affinities of −5.1 and −4.9 kcal/mol. Still, these showed hydrogen bonds with two critical amino acid residues of the enzyme activity, GLU 233 and ASP 300. The findings of the molecular docking analysis of compounds of ethyl acetate extract of *C*. *comosum* leaf with α-amylase indicated that fumaric acid, 2-methoxyphenyl 2,3-dichlorophenyl ester has the highest binding affinity of −8.8 kcal/mol with the enzyme in comparison with other compounds. However, fumaric acid, 2-methoxyphenyl 2,3-dichlorophenyl ester, didn’t form any conventional hydrogen bond with the crucial amino acid residues associated with the enzyme activity. Heptadecanoic acid bound with α-amylase with a lower binding affinity of −4.9 kcal/mol, but it formed hydrogen bonds with GLU 233 and ASP 300, two of the crucial amino acid residues of the enzyme activity. Meanwhile, the standard acarbose formed a hydrogen bond with only one of the critical amino acid residues of the α-amylase activity, ASP197, with a binding affinity of −7 kcal/mol.

**TABLE 6:** Binding affinity of docked phytochemical compounds of ethyl acetate extract of *Chlorophytum comosum* root with α-amylase and α-glucosidase.

Phytocompounds	Binding energy (Kcal/mole)	
	α-amylase	α-glucosidase
Benzoic acid	−5.1	−5.6
Benzene, 1-(1,5-dimethyl-4-hexenyl)-4-methyl-	−7.1	−7.6
2,4-Di-tert-butylphenol	−6.4	−7.5
Dodecanoic acid	−5.1	−5.9
2,2-Dimethyl-2-[2.3,5,6-tetramethyl]ethanol	−6.6	−5.7
Phenol, 5-(1,5-dimethyl-4-hexenyl)-2-methyl-, (R)-	−7.4	−7.7
Tetradecanoic acid	−5.5	−6.4
Methyl 2-methoxy-1-naphthoate	−6.3	−6.4
Ethanone, 1.1’-(6-hydroxy-2,5-benzofurandiyl)bis-	−6.9	−7.8
Pentadecanoic acid	−4.9	−6.6
3,5-Di-tert-butyl-2-hydroxybenzaldehyde	−6.4	−7.5
(E)-3-(2-Methoxyphenyl)-2-propenoic acid	−5.9	−6.8
Methyleugenol	−5.4	−6.1
7,9-Di-tert-butyl-1-oxaspiro (4,5)deca-6,9-diene-2,8-dione	−6.5	−7.4
Palmitoleic acid	−5	−6.6
n-Hexadecanoic acid	−5.2	−6.3
1-Eicosene	−5.2	−6.2
Benzoic acid, 4-heptyl-, 4-cyanophenyl ester	−6.6	−8.1
cis-10-Heptadecenoic acid	−5.2	−6.8
Heptadecanoic acid	−4.9	−6.5
1-Octadecene	−5.2	−6.2
2-Nonadecanone	−4.8	−6.3
9(E),11(E)-Conjugated linoleic acid	−5.1	−6.6
Oleic Acid	−5.4	−6.5
Octadecanoic acid	−4.8	−6.6
Hexacosane	−5.1	−5.5
1,1-Biphenyl, 2,3,4,4′-tetramethoxy-5-methyl-6′-methoxycarbonyl-	−6.8	−6.2
Nonacosane	−5	−5.3
α-Tocopherol	−6.9	−7.6
Campesterol	−8.8	−3.7
Stigmasterol	−9.4	−7.9
Ursa-9 (11),12-dien-3-ol	−10	10.7
γ-Sitosterol	−9.1	−7.3
Lup-20 (29)-en-3-one	−9.6	8.9
Stigmast-4-en-3-one	−8.7	−4.5
Pennogenin	−9	−1.3
Acarbose	−7	−6

**TABLE 7:** Binding affinity of docked phytochemical compounds of ethyl acetate extract of *Chlorophytum comosum* leaf with α-amylase and α-glucosidase.

Phytocompounds	Binding energy (Kcal/mole)	
	α-amylase	α-glucosidase
Benzene, 1-(1,5-dimethyl-4-hexenyl)-4-methyl-	−7.1	−7.6
2,4-Di-tert-butylphenol	−6.4	−7.5
Phenol, 5-(1,5-dimethyl-4-hexenyl)-2-methyl-, (R)-	−7.4	−7.7
1-Octadecene	−5.2	−6.2
n-Hexadecanoic acid	−5.2	−6.3
1-Eicosene	−5.2	−6.2
Heptadecanoic acid	−5.1	−6.3
Kaur-16-ene	−8.7	−4.6
Phytol	−5.8	−7.4
9,12,15-Octadecatrienoic acid, (Z,Z,Z)-	−6.1	−7.1
Octadecanoic acid	−4.8	−6.6
28-Norolean-17-en-3-one	−9.7	−3.4
1,2-Cyclohexanedicarboxylic acid, dodecyl 2-ethoxyethyl ester	−5.4	−5.9
α-Tocopherol	−6.9	−7.6
Stigmasterol	−9.4	−7.9
γ-Sitosterol	−9.1	−7.3
Fumaric acid, 2-methoxyphenyl 2,3-dichlorophenyl ester	−6.9	−8.8
Benzo [h]quinoline, 2,4-dimethyl-	−7.6	−7.1
Octacosanoic acid, ethyl ester	−5.1	−6.3
Acarbose	−7	−6

Molecular docking analysis of compounds of ethyl acetate extract of *C*. *comosum* root with α-glucosidase indicated that benzoic acid, 4-heptyl-, 4-cyanophenyl ester has a higher binding affinity of −8.1 kcal/mol with the protein structure compared to the other compounds. However, benzoic acid, 4-heptyl-, 4-cyanophenyl ester did not form any hydrogen bond with the amino acid residues of the enzyme activity. The compound, ethanone, 1,1’-(6-hydroxy-2,5-benzofurandiyl)bis-bound with α-glucosidase enzyme with a comparatively lower binding affinity of −7.8 kcal/mol, but it showed hydrogen bonds with two of the crucial amino acid residues of the enzyme activity, GLU 271 and ASP 333. Heptadecanoic acid and dodecanoic acid bound with α-glucosidase with binding affinities of −6.5 and −5.9 kcal/mol and formed a hydrogen bond with ASP 333, one of the critical amino acid residues of the enzyme activity. Molecular docking analysis of compounds of ethyl acetate extract of *C*. *comosum* leaf with α-glucosidase indicated that fumaric acid, 2-methoxyphenyl 2,3-dichlorophenyl ester has a higher binding affinity of −8.8 kcal/mol with the protein structure in comparison to other compounds present. However, fumaric acid, 2-methoxyphenyl 2,3-dichlorophenyl ester, didn’t establish any hydrogen bond with the amino acid residues of the enzyme activity. The compounds phenol, 5-(1,5-dimethyl-4-hexenyl)-2-methyl-, benzo [h]quinoline, 2,4-dimethyl-, and heptadecanoic acid bound with α-glucosidase with comparatively lower binding affinities of −7.7, −7.1, and −6.3 kcal/mol respectively, but these formed hydrogen bonds with one of the critical amino acid residues of the enzyme activity, ASP 333, ASP 333, and GLU 271 respectively. Despite its lower binding affinity of −6 kcal/mol, the standard, acarbose, interacted with GLU271 and ASP333 through hydrogen bonding. Hyperglycemia can be controlled by inhibiting the carbohydrate hydrolysing enzymes α-amylase and α-glucosidase, which prevents the hydrolysis of carbohydrates into glucose molecules (Kato-Schwartz et al., 2020). The ability of heptadecanoic acid and dodecanoic acid to form hydrogen bonds with amino acid residues in the active sites of α-amylase and α-glucosidase enzymes, compared to the standard drug, acarbose indicates their potential as inhibitors of α-amylase and α-glucosidase, which aids in managing hyperglycemia. So, the mechanism of the hypoglycemic property of heptadecanoic acid and dodecanoic acid is proposed to inhibit glucose absorption (Mechchate et al., 2021).

#### ADMET analysis of heptadecanoic acid and dodecanoic acid

**FIGURE 7:**
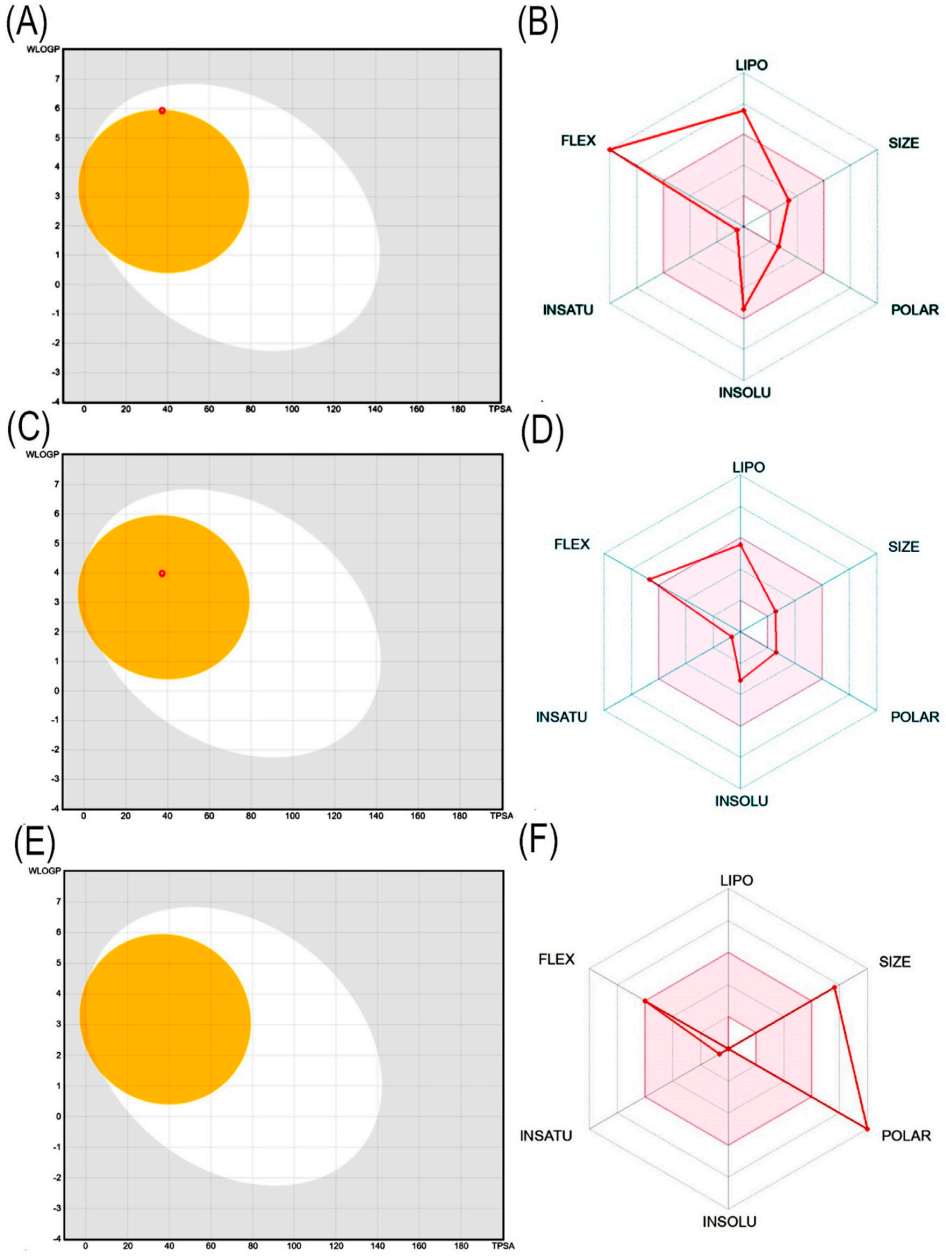
**(A)** Boiled egg graphical representation of heptadecanoic acid, **(B)** Bioavailability radar of heptadecanoic acid, **(C)** Boiled egg graphical representation of dodecanoic acid, **(D)** Bioavailability radar of dodecanoic acid, **(E)** Boiled egg graphical representation of acarbose, **(F)** Bioavailability radar of acarbose.

The most potential antidiabetic bioactive compounds of ethyl acetate extracts of *C*. *comosum* roots and leaves identified through molecular docking were analysed for ADMET properties (Tables 8, 9). The ADMET analysis utilizes a set of rules based on knowledge, which includes analysing the compounds’ bioavailability, solubility, gastrointestinal absorption (GIA), blood-brain barrier (BBB) permeability, molecular weight (MW), molar refractivity (MR), topological polar surface area (TPSA), hydrogen bond acceptors (HBA), hydrogen bond donors (HBD), Cytochrome P450 (CYP) inhibition and toxicity. TPSA influences the movement of molecules across biological membranes. Typically, compounds with a TPSA of less than 140 Å^2^ can easily and quickly permeate through membranes, resulting in fewer adverse side effects (Kibet et al., 2024). Both heptadecanoic acid and dodecanoic acid have 37.30 Å^2^ TPSA. This suggests that these compounds have high permeability and bioavailability and fewer adverse side effects (Vulichi et al., 2023). The standard drug, acarbose, had a TPSA value of 321.17 Å^2^, which indicates its less permeability through biological membranes and adverse side effects. The log P value is a measure of a compound’s lipophilicity or hydrophobicity. A log p value exceeding 5 indicates that the compound is highly lipophilic, which can lead to high toxicity since the drug remains in the lipid bilayer membrane for an extended period and spreads throughout. Conversely, the negative value of log p indicates a hydrophilic compound, making it difficult for the compound to be absorbed (Lobo and Lobo, 2020). A compound can be considered to have good absorption and permeation qualities if its log P value is ≤5 and not negative, and this enables the compound to pass through the hydrophilic outer layer of the membrane and gain access to the hydrophobic lipid bilayer. The Log p values of both compounds were in the acceptable range, i.e., < 5, suggesting the compounds’ bioavailability for an oral dose (Molla and Aljahdali, 2023). The log p value of acarbose was also in the acceptable range. The Log S value of −5.37 indicates that heptadecanoic acid is moderately soluble in water, and the Log S value of −3.07 indicates that dodecanoic acid is soluble in water. The Log S value of 2.13 indicated that acarbose is soluble in water. These compound’s MR was also in the acceptable range, 40–130, whereas the MR of acarbose was 136.69. Drugs’ absorption in the gastrointestinal tract is a critical factor in their distribution throughout the body. Once drugs have been absorbed, they are then dispersed throughout the body. After absorption, drugs need to penetrate the blood-brain barrier to reach the central nervous system. Hyperglycemia can affect the transport function through the BBB. Changes in the transport function include alterations in glucose, insulin, choline, and amino acid transport. In addition, hyperglycemia can compromise the integrity of the blood-brain barrier by disrupting tight junctions and causing oxidative stress in the microcapillaries of the central nervous system (Mateusz et al., 2023). Heptadecanoic acid and dodecanoic acid show high GIA and cross the BBB, compared to the standard drug, acarbose, which has low GIA and doesn’t cross the BBB. The boiled egg graphical representations of heptadecanoic acid, dodecanoic acid, and acarbose were analysed using the swissADME tool (Figures 7A, C, E). The egg white region displays GIA, the egg yellow region displays penetration of the BBB, and the egg grey region displays different routes in addition to the oral route. It was deduced from the boiled egg graphs that the compounds have high GIA and can permeate the BBB. Drug likeness evaluates a compound’s bioavailability as a potential oral drug (Raut et al., 2023). The bioavailability radar of heptadecanoic and dodecanoic acid shows its drug-likeness characteristics (Figures 7B, D, F). The pink portion of the radar indicates the required range to access the drug-likeness trait. The HBD and HBA centers assist in forming H-bonds and improve the solubility of water. Heptadecanoic acid followed Lipinski’s rule, with a MW of 270.5 g/mol, 4.11 log p, 1HBD, and 2 HBAs, showing a drug-likeness characteristic (Paul and Majumdar, 2023). Dodecanoic acid followed Lipinski’s rule, with 200.32 g/mol MW, 2.70 log p, 1 HBD, and 2 HBAs. Heptadecanoic acid followed Lipinski’s rule with one violation, and dodecanoic acid followed Lipinski’s rule without any violations, compared to acarbose, which didn’t follow Lipinski’s rule and exhibited 3 violations. Acarbose had a MW of 645.60 g/mol, which is >500 g/mol, which indicates its less permeability through biological membranes. Acarbose also had 14 HBD, which is >5, and 19 HBAs, which is >10, and thereby deviates from the acceptable range. CYP refers to a cluster of isozymes involved in the metabolism of drugs, fatty acids, steroids, bile acids, and carcinogens (Esteves et al., 2021). Predicted values indicate that both heptadecanoic and dodecanoic acid are neither substrates nor inhibitors of CYP enzymes. This suggests that these compounds are likely to undergo efficient metabolism in the liver and be readily excreted from the body, thus minimizing the probability of causing toxic effects. The results significantly support the potential of the bioactive compounds to function as drugs. The standard drug, acarbose, was also a non-inhibitor of CYP enzymes. The compound’s bioactivity scores (BAS) for drug targets have also been predicted. In general, a higher BAS indicates a greater likelihood of the studied compound being active. A compound with a BAS greater than 0.00 is expected to exhibit substantial biological activities, while values ranging from −0.50 to 0.00 are considered moderately active, and those below −0.50 are assumed to be inactive (Erdoğan et al., 2023). Both heptadecanoic acid and dodecanoic acid had a BAS of 0.85, compared to acarbose, which had a lower BAS of 0.17. It is suggested that these compounds are biologically active, have high bioavailability, and have a greater likelihood of medicinal effects. Toxicology is an important factor that affects how a drug is absorbed, distributed, metabolized, and excreted in the body (Gaohua et al., 2021). Toxicology profiling includes assessing human hepatotoxicity, mutagenicity, drug-induced liver injury, skin sensitization, and median lethal dose (LD_50_) of acute toxicity. In clinical trials, if a drug causes liver damage, it can lead to discontinuation of development efforts, which is inconvenient and expensive. Drug-induced liver damage is a major concern for patient safety and can lead to medications being withdrawn from the market (William et al., 2021). Both heptadecanoic acid and dodecanoic acid are less harmful to the liver, as indicated by the predicted hepatotoxic probability values. Mutagenicity testing is widely used to determine the potential of substances to cause mutations (Saeed et al., 2022). The predicted mutagenic probability values indicate the low mutagenicity/toxicity of compounds. Assessing acute toxicity in animals is crucial in drug safety evaluation (Movia et al., 2020). The acute oral toxicity values for both compounds were within acceptable ranges. A significant safety concern is determining if a substance that comes into contact with the skin can cause allergic contact dermatitis (Saeed et al., 2022). Products applied to the skin can lead to skin sensitization. However, the compounds were found to be non-skin sensitive. The standard drug, acarbose, was also found to be non-hepatotoxic, non-mutagenic, non-skin sensitive, and less toxic, but it is found to induce liver injury. Both heptadecanoic acid and dodecanoic acid have the ideal physicochemical properties required for drug-likeness and less toxicity and also follow Lipinski’s rule, which indicates that the molecular structure of these compounds is suitable for drug design, safety, and bioavailability as a potential oral drug (Raut et al., 2023).

**TABLE 8:** ADME properties of heptadecanoic acid and dodecanoic acid.

	MW(g/mol)	HBD	HBA	MR	TPSA (Å^2^)	Log *S*	LogP_o/w_	GIA	BBB permeant	Lipinski’s (violations)	CYP3A4 inhibitor	BAS
Heptadecanoic acid	270.5	1	2	85.60	37.30	−5.37	4.11	High	Yes	Yes (1)	No	0.85
Dodecanoic acid	200.32	1	2	61.57	37.30	−3.07	2.70	High	Yes	Yes (0)	No	0.85
Acarbose	645.60	14	19	136.69	321.17	2.13	1.43	Low	No	No (3)	No	0.17

**TABLE 9:** Toxicity analysis of heptadecanoic acid and dodecanoic acid.

	Human hepatotoxicity	Ames mutagenicity	Drug-induced liver injury	Skin sensitization	LD_50_ of acute toxicity
Heptadecanoic acid	0.092	0.008	0.356	0.115	>500 mg/kg
Dodecanoic acid	0.18	0.008	0.356	0.115	>500 mg/kg
Acarbose	0.418	0.256	0.678	0.236	>500 mg/kg

### *In vitro* hypoglycemic potential of ethyl acetate extracts of *C. comosum* root and leaf

#### α-amylase and α-glucosidase inhibition activity

**FIGURE 8:**
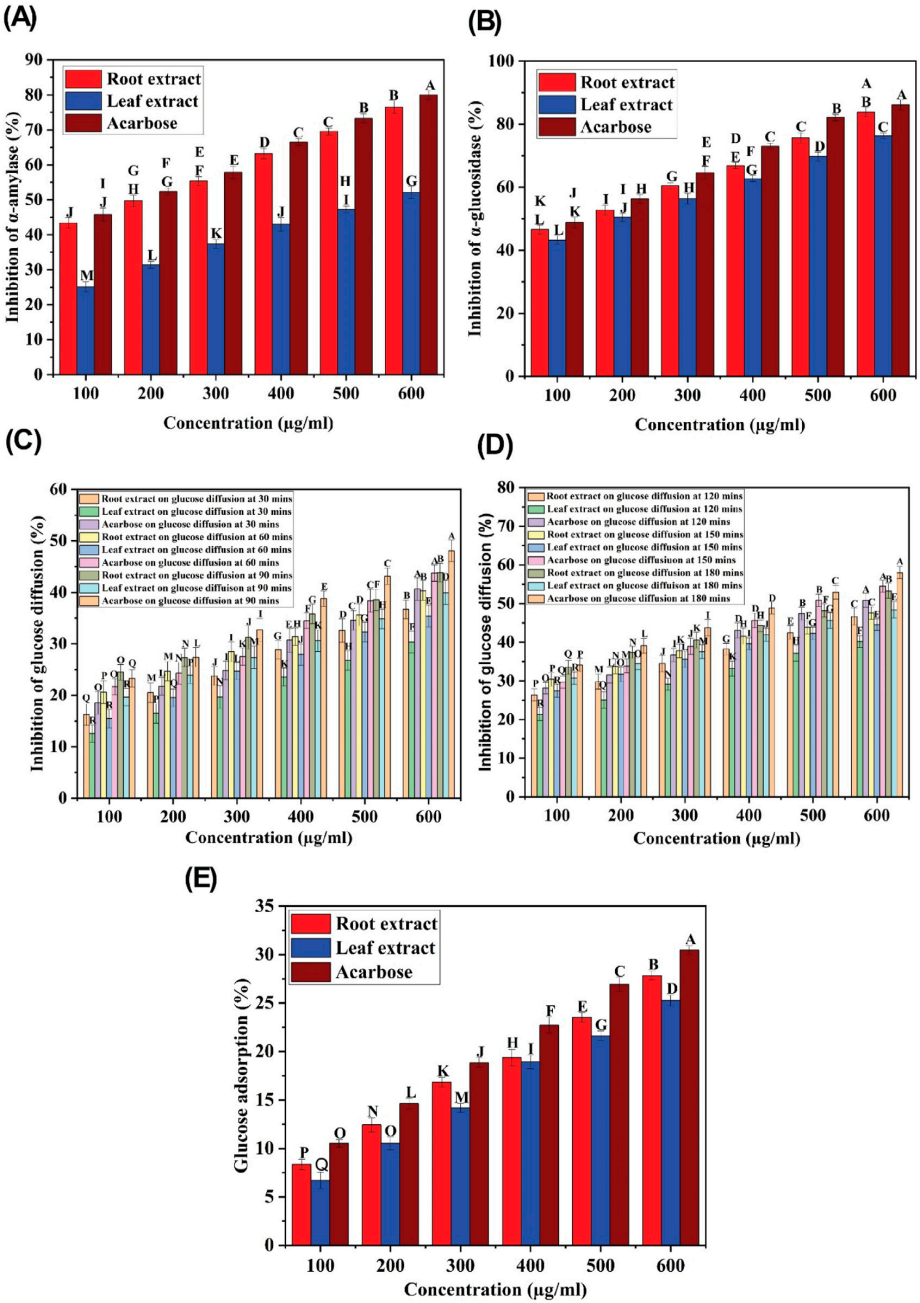
**(A)** α-amylase inhibitory activity of ethyl acetate extracts of *C. comosum* root and leaf, **(B)** α-glucosidase inhibitory activity of ethyl acetate extracts of *C. comosum* root and leaf, **(C)** Glucose diffusion inhibitory activity of ethyl acetate extracts of *C. comosum* root and leaf at 30, 60, and 90 min, **(D)** Glucose diffusion inhibitory activity of ethyl acetate extracts of *C. comosum* root and leaf at 120, 150, and 180 min, **(E)** Glucose adsorption capacity of ethyl acetate extracts of *C. comosum* root and leaf. Different letters indicate significant differences between the tested sample and standard (*p* < 0.05).

The inhibition of α-amylase and α-glucosidase enzymes, which hydrolyze carbohydrates into glucose molecules and cause the elevation of blood glucose levels, is a promising therapeutic approach to managing hyperglycemia (Gök et al., 2020; Sansenya et al., 2020). The ethyl acetate extracts of *C. comosum* root and leaf exhibited higher inhibitory potential on α-glucosidase than α-amylase, as indicated by the percentage of inhibition. The α-amylase and α-glucosidase inhibition activity of ethyl acetate extracts of *C. comosum* root and leaf was concentration-dependent (Figures 8A, B). The inhibitory effect improved as the concentration of the extracts increased from 100 to 600 μg/mL. The inhibition percentage of the ethyl acetate extract of *C*. comosum root was approximately 0.319 times higher on α-amylase and 0.089 times higher on α-glucosidase than the ethyl acetate extract of *C. comosum* leaf. The inhibitory activity of the ethyl acetate extracts of *C. comosum* root and leaf on α-amylase and α-glucosidase was comparable with the inhibition percentages based on the IC_50_ values (Table 10). Ethyl acetate extract of *C. comosum* root had higher inhibitory activity on α-glucosidase with an IC_50_ value of 179.34 ± 0.3 μg/mL, and the inhibition percentage increased to 83.8 ± 1.55 when the concentration of the extract reached 600 μg/mL. In comparison, it exhibited comparatively lesser inhibitory activity on α-amylase with an IC_50_ value of 205.39 ± 0.15 μg/mL. The ethyl acetate extract of *C. comosum* leaf inhibited α-amylase and α-glucosidase enzymes with IC_50_ values of 547.99 ± 0.09 and 198.18 ± 0.25 μg/mL, respectively. The standard acarbose showed IC_50_ values of 167.46 ± 0.13 and 111.48 ± 0.18 μg/mL on α-amylase and α-glucosidase respectively. Ethyl acetate extracts of *C. comosum* root and leaf inhibit α-amylase and α-glucosidase enzymes, therefore inhibiting the release of glucose from carbohydrates, slowing down glucose absorption, and reducing postprandial hyperglycemia. Heptadecanoic acid and dodecanoic acid were identified as the potential compounds with α-amylase and α-glucosidase inhibitory activities through molecular docking studies. This suggests that these compounds support the postprandial blood glucose-lowering ability of the ethyl acetate extracts of *C*. *comosum* root and leaf by preventing the breakdown of carbohydrates into glucose molecules.

**TABLE 10:** Inhibitory potential of ethylacetate extracts of *C. comosum* roots and leaves on α-amylase, α-glucosidase, and glucose diffusion.

Samples	Inhibition at 600 μg/mL (%)			IC_50_ values (µg/mL)		
	α-amylase	α- glucosidase	Glucose diffusion	α-amylase	α- glucosidase	Glucose diffusion
Root extract	76.48 ± 1.81	83.8 ± 1.55	53.25 ± 1.9	205.39 ± 0.15	179.34 ± 0.3	535.248
Leaf extract	52.1 ± 1.79	76.33 ± 0.89	48.38 ± 2.1	547.99 ± 0.09	198.18 ± 0.25	-
Acarbose	80 ± 1.26	86.12 ± 1.58	58.02 ± 1.6	167.46 ± 0.13	111.48 ± 0.18	432.07 ± 0.95

Note: The values are represented as the standard deviation of the mean (mean ± SD), n = 3, *p* < 0.05, Abbreviation: IC_50_, half maximal inhibitory concentration.

#### Glucose diffusion inhibition activity

Several decades of research have reported the beneficial effects of plant fiber in regulating blood glucose levels (Gallagher et al., 2003). In this study, an *in vitro* dialysis membrane-based assay was conducted to investigate how varying concentrations of ethyl acetate extracts of *C. comosum* root and leaf affect glucose diffusion. The glucose movement across the membrane was monitored at intervals of 30 min, i.e., from 30 min to 180 min. The diffusion of glucose across the dialysis membrane increased between 30 and 180 min. The ethyl acetate extracts of *C. comosum* root and leaf significantly inhibited the glucose movement into the external solution in a dose-dependent manner, as displayed in Figures 8C, D. The ethyl acetate extract of *C. comosum* root retarded diffusion of glucose more compared to the leaf extract with the highest inhibition percentage of 53.25 ± 1.9 at 600 μg/mL concentration. The ethyl acetate extract of *C*. comosum root was approximately 0.0914 times more effective in inhibiting glucose diffusion than the leaf extract. The maximum inhibition percentage showed by the ethyl acetate extract of *C*. *comosum* leaf was 48.38 ± 2.1 at 600 μg/mL concentration. However, the ethyl acetate extract of *C. comosum* root was less effective than acarbose in inhibiting glucose diffusion by approximately 0.0819 times. Ethyl acetate extract of *C. comosum* root inhibited glucose diffusion with an IC_50_ value of 535.248 μg/mL, while acarbose had an IC_50_ value of 432.07 ± 0.95 μg/mL (Table 10). The presence of fiber in the plant extract is capable of impeding the movement of glucose, thereby reducing its adsorption in the digestive tract (Gallagher et al., 2003; Paul and Majumdar, 2023).

#### Effect on glucose adsorption

The effect of the ethyl acetate extracts of *C. comosum* root and leaf on glucose adsorption has been shown in Figure 8E. The results indicate that the extracts have a significant capability to adsorb glucose at all tested concentrations. A direct correlation between the extracts’ ability to adsorb glucose and the molar concentration of glucose was observed, with lower adsorption at 5 mM and higher adsorption at 25 mM concentration of glucose. The extracts’ capability to adsorb glucose may be attributed to their components, as both soluble and insoluble parts and fibers from other plant sources are known to adsorb glucose. The amount of glucose in the small intestine can be restricted by the glucose adsorption property, resulting in a decreased amount of glucose being transported through the digestive tract, thus impeding glucose absorption (Chau et al., 2004; Bhutkar et al., 2017). Even at lower concentrations of glucose (5 mM), the ethyl acetate extracts of *C. comosum* root and leaf were able to bind glucose, thereby reducing the amount of glucose available for transport in the digestive tract and mitigating postprandial hyperglycemia. The glucose adsorption capacity of the ethyl acetate extract of *C. comosum* root was approximately 0.0919 times higher than that of the leaf extract. However, the ethyl acetate extract of *C. comosum* root was less effective than acarbose on glucose adsorption by approximately 0.0869 times.

### *In vitro* antioxidant potential of ethyl acetate extracts of *C. comosum* root and leaf

**FIGURE 9:**
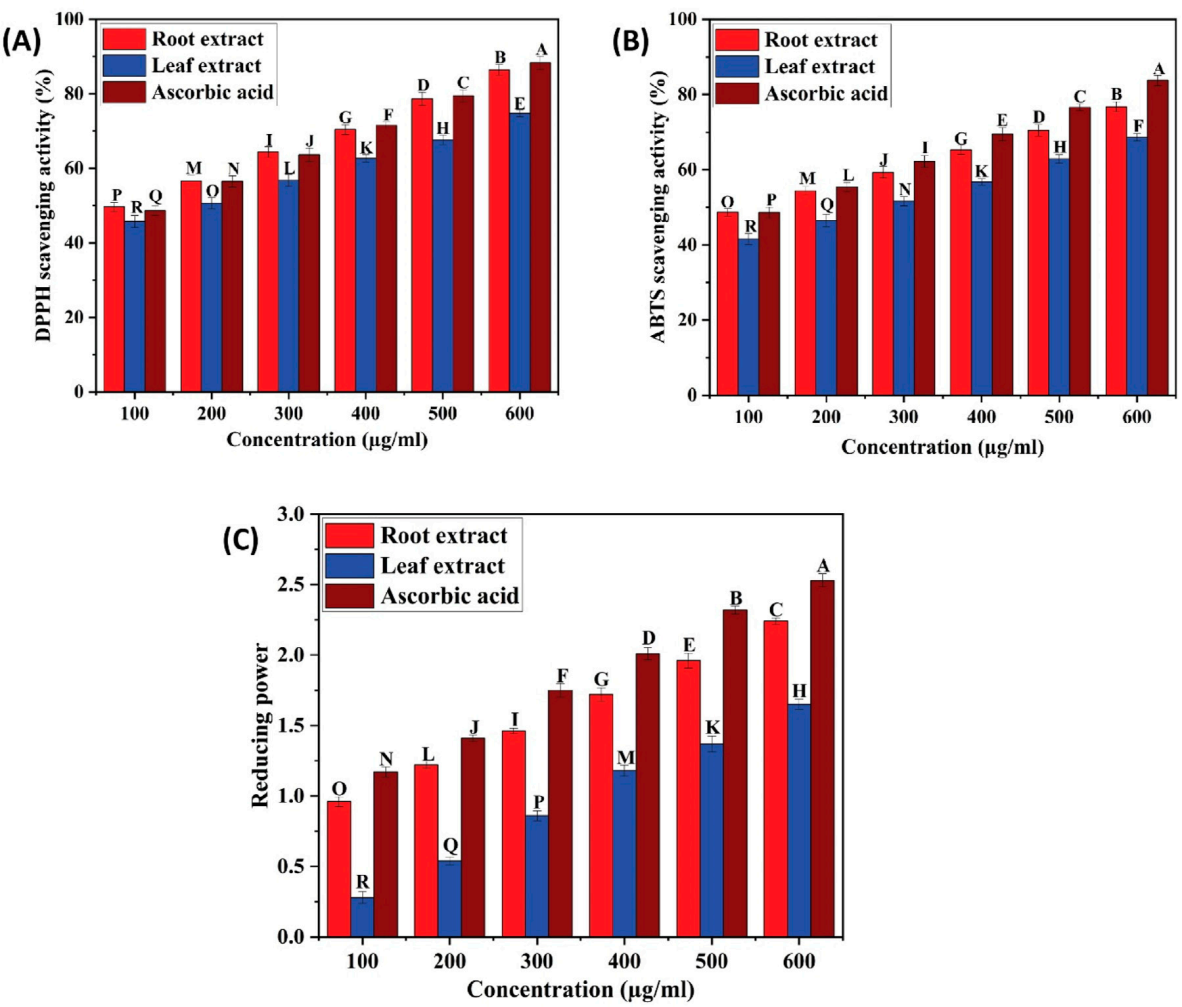
**(A)** DPPH scavenging activity of ethyl acetate extracts of *C. comosum* root and leaf, **(B)** ABTS scavenging activity of ethyl acetate extracts of *C. comosum* root and leaf, **(C)** Reducing ability of ethyl acetate extracts of *C. comosum* root and leaf. Different letters indicate significant differences between the tested sample and standard (*p* < 0.05).

The effectiveness of plant extracts as antioxidants stems from their ability to act as reducing agents due to their redox properties. This capacity is generally attributed to the presence of reductants that carry out antioxidant functions by either donating a hydrogen atom to interrupt the free radical chain or by inhibiting peroxide formation. Medicinal plant tissues are abundant in phenolic compounds, which possess a wide array of biological effects, including antioxidant properties. The antioxidant potential of ethyl acetate extracts of *C. comosum* root and leaf was evaluated using DPPH, ABTS, and FRAP assays, and the results are given in Table 11; Figures 9A–C. DPPH assay has been commonly employed to assess the antioxidant activity of plant extracts by scavenging free radicals, which results in a color change from purple to yellow (Fu et al., 2021; Globularia et al., 2021). Ethyl acetate extracts of *C. comosum* root and leaf exhibited effective DPPH scavenging activity in a concentration-dependent manner with IC_50_ values of 108.37 ± 0.06 and 181.79 ± 0.09 µM, respectively. Ethyl acetate extract of *C. comosum* root exhibited the maximum scavenging activity and was 86.46% ± 1.45% at the concentration of 600 µM. The standard ascorbic acid showed the scavenging effect on DPPH free radical with an IC_50_ value of 121.105 ± 0.08 µM. Ethyl acetate extract of *C. comosum* root had higher scavenging activity on DPPH than the leaf extract, around 0.136 times. ABTS assay is frequently employed to assess the total antioxidant activity of plant extracts (Extracts et al., 2018; Tiji et al., 2021). The total antioxidant capacity of the extracts was assessed by eliminating ABTS free radicals, which resulted in a color change from green/blue to yellow. The extracts’ concentration and ability to scavenge free radicals in ABTS exhibit a dose-dependent relationship. The ABTS scavenging activity of the ethyl acetate extracts of *C. comosum* root and leaf increases from 48.64% ± 0.99% to 76.73% ± 1.35% and 41.54% ± 1.42% to 68.64% ± 0.99%, respectively, when its concentration ranges from 100–600 µM. Hence, the ABTS free radical scavenging effects of *C. comosum* root and leaf ethyl acetate extracts depend highly on concentration. Ethyl acetate extract of *C. comosum* root exhibited the highest scavenging activity and was 76.73% ± 1.35% with an IC_50_ value of 126.24 ± 0.13 µM. The standard ascorbic acid exhibited the scavenging effect on ABTS free radical with an IC_50_ value of 123.049 ± 0.11 µM. Ethyl acetate extract of *C. comosum* root had higher scavenging activity on ABTS than the leaf extract, around 0.105 times. The capacity to function as a reductant is a significant factor in assessing antioxidant activity (Fu et al., 2021). Ethyl acetate extracts of *C. comosum* root and leaf showed the ability to reduce the ferric ion complex, tripyridyltriazine (TPTZ), i.e., the reduction of the ferricyanidecomplex to ferrocyanide, which forms an intense blue Fe^2+^ - TPTZ complex. There is a strong correlation between the extracts’ reducing ability and concentration. With the increase of concentration of the ethyl acetate extracts of *C. comosum* root and leaf (100–600 µM), the reducing power increases from 0.96 ± 0.03 to 2.24 ± 0.02 and 0.28 ± 0.04 to 1.65 ± 0.03 respectively. Ethyl acetate extract of *C. comosum* root had the maximum reducing power and was 2.24 ± 0.02 at 600 µM concentration. The maximum reducing power showed by the standard ascorbic acid was 2.53 ± 0.04. Ethyl acetate extract of *C. comosum* root had higher reducing power than the leaf extract, around 0.263 times. The antioxidant activity of ethyl acetate extracts of *C. comosum* root and leaf may be attributed to the presence of fatty acids, 9(E), 11(E)-Conjugated linoleic acid and n-Hexadecanoic acid respectively, which are regarded as strong antioxidants and are identified as major compounds of the extracts through GCMS analysis (Saha and Ghosh, 2012; Singh et al., 2022; Ganesan et al., 2024).

**TABLE 11:** Free radical scavenging activity and reducing power of ethyl acetate extracts of *C. comosum* root and leaf.

	Scavenging activity at 600 µM (%)		Reducing power	IC_50_ values (µM)	
	DPPH	ABTS		DPPH	ABTS
Root extract	86.46 ± 1.45	76.73 ± 1.35	2.24 ± 0.02	108.37 ± 0.06	126.24 ± 0.13
Leaf extract	74.72 ± 0.89	68.64 ± 0.99	1.65 ± 0.03	181.79 ± 0.09	264.409 ± 0.08
Ascorbic acid	88.34 ± 1.77	83.74 ± 1.37	2.53 ± 0.04	121.105 ± 0.08	123.049 ± 0.11

Note: The values are represented as the standard deviation of the mean (mean ± SD), n = 3, *p* < 0.05, Abbreviation: IC_50_, half maximal inhibitory concentration.

## Conclusion

This is the first study to evaluate the *in vitro* and *in silico* hypoglycemic potential of *C*. *comosum* root and leaf ethyl acetate extracts. The ethyl acetate extract of *C. comosum* root has higher inhibitory potential on α-amylase, α-glucosidase, and glucose diffusion, and enhanced glucose adsorption than the ethyl acetate extract of *C. comosum* leaf. The ethyl acetate extract of *C. comosum* root also exhibited promising reducing power capability and scavenging activity against DPPH and ABTS free radicals *in vitro* than the ethyl acetate extract of *C. comosum* leaf. Molecular docking analysis supported the hypoglycemic potential of ethyl acetate extracts of *C. comosum* root and leaf. Heptadecanoic acid and dodecanoic acid were identified as potential compounds with α-amylase and α-glucosidase inhibitory activities through *in silico* molecular docking analysis. Based on the *in silico* ADMET analysis, heptadecanoic acid and dodecanoic acid have the ideal physicochemical properties required for drug-likeness, high bioavailability, and less toxicity. This suggests that these compounds are suitable for drug design. Further studies are needed to isolate the potential compounds and elucidate their mechanism of action in managing hyperglycemia. Ethyl acetate extracts of *C. comosum* root and leaf can be appraised as a natural source of hypoglycemic and antioxidant properties, suggesting future applications in developing antihyperglycemic drugs.

## Data Availability

The original contributions presented in the study are included in the article/supplementary material, further inquiries can be directed to the corresponding author.

## Abbreviations

ADMET, Absorption, Distribution, Metabolism, Excretion, and Toxicity; ABTS, 2.2′-azinobis (3-ethylbenzothiazoline-6-sulfonic acid; BAS, Bioactivity score; BBB, Blood-brain barrier; *C. comosum*, *Chlorophytum comosum*; CYP, Cytochrome P450; FRAP, Ferric reducing antioxidant power; FC, Folin-Ciocalteu; FTIR, Fourier transform infrared spectroscopy; GAE, Gallic acid equivalent; GC-MS, Gas chromatography-mass spectrometry; GIA, Gastrointestinal absorption; GOD-POD, Glucose oxidase-peroxidase; IC_50_, Half maximal inhibitory concentration; HBA, hydrogen bond acceptor; HBD, hydrogen bond donor; LD_50_, Median lethal dose; MR, Molar refractivity; MW, Molecular weight; NIST, National Institute of Standards and Technology; DPPH, 2,2-diphenyl-1-picrylhydrazyl; PDB, Protein data bank; TPSA, Topological polar surface area; TPTZ, Tripyridyltriazine; TFC, Total flavonoid content; TPC, Total phenolic content; T2DM, Type 2 diabetes mellitus; QUE, Quercetin equivalent.
